# Lightweight Fault Identification of Magnetic Latching Needle Selectors Based on Multistage Transient Drive Current Responses

**DOI:** 10.3390/s26216891

**Published:** 2026-10-30

**Authors:** Laihu Peng, Chenyang Zhu, Yubao Qi, Shuo Yang, Kaiyuan Shao

**Affiliations:** 1 Zhejiang Key Laboratory of Intelligent Manufacturing Equipment for Flexible Functional Materials, Faculty of Mechanical Engineering and Automation, Zhejiang Sci-Tech University, Hangzhou 310018, China; laihup@zstu.edu.cn (L.P.); 2024220503246@mails.zstu.edu.cn (C.Z.); 2025220503227@mails.zstu.edu.cn (S.Y.); 2 Modern Textile Technology Innovation Center (Jianhu Laboratory), Shaoxing 312000, China; 3 Rifa Textile Machinery Research Institute, Zhejiang Rifa Textile Machinery Co., Ltd., No. 88 Woxi Avenue, Meizhu Industrial Zone, Shaoxing 312532, China; shaoky@rifagroup.com

**Keywords:** magnetic latching needle selector, transient drive current, multi-stage response, fault identification, lightweight network, online state monitoring

## Abstract

Magnetic latching needle selectors are key actuating components that perform needle selection in circular knitting machines, and their operating states directly affect weaving reliability. To address the difficulty of online fault monitoring during large-scale deployment of needle selectors, a lightweight fault identification method based on multi-stage transient drive current response is proposed. An electromechanical coupling model is established, and single-factor perturbation analysis is used to reveal the three-stage transient current response and its variations with key parameters. Using measured currents from the normal state and five fault states, 14-dimensional features are constructed to describe stage macro characteristics, local dynamics, and equivalent electromagnetic responses. A Gated Low-Rank Cross Network (GLR-CrossNet) is then used for six-state identification. Across ten specimen-level data partitions, the proposed method achieved an Accuracy of 95.54 ± 0.55% and a Macro-F1 score of 95.55 ± 0.56%. After deployment on the needle selector control terminal, the end-to-end identification time is 3.11 ms, while continuous operation tests on a circular knitting machine achieve a single-action accuracy of 94.20% for the five fault states. The results support lightweight online state monitoring of magnetic latching needle selectors using transient drive current.

## 1. Introduction

The magnetic latching needle selector is a key actuator that performs needle selection in computerized jacquard circular weft knitting machines. During knitting, the selector blade must reverse frequently according to pattern commands and reliably reach the corresponding limit position. The dynamic response of the needle selection mechanism is affected by machine operating conditions, mechanical parameters, and drive performance [1,2,3], while baffle wear, foreign object blockage, changes in component position, and electromagnetic degradation may cause premature, delayed, or incomplete selector blade actuation, thereby affecting normal needle selection. Circular weft knitting machines typically use a large number of needle selectors, and if abnormalities at individual blade positions are not detected in time, they can lead to incorrect or missed needle selection during continuous knitting. Timely acquisition of needle selector operating conditions is important for fault localization and equipment maintenance.

Existing condition monitoring methods for magnetic latching needle selectors mainly rely on external information such as selector blade motion and knitting needle operating conditions. Li et al. [4] designed a needle selector actuation frequency detection system based on the stroboscopic principle and obtained the motion state of the selector blade through periodic optical observation, enabling detection of the needle selection process. Zhang et al. [5] combined laser detection with machine vision to identify abnormal conditions of knitting needles in hosiery machines and determined their operating conditions from positional changes and abnormal features during needle motion. Yuan et al. [6] used a laser displacement sensor to measure displacement changes and response time during selector blade motion in an electromagnetic needle selector, enabling detection of needle selector actuation performance. Image processing methods have also been used to analyze changes in the limit position of the selector blade and abnormal motion conditions [7]. These methods can directly obtain the actuation condition of the needle selector, but usually require additional optical, vision, or displacement sensing devices. However, circular weft knitting machines contain a large number of needle selectors within limited installation space, making large scale deployment of external detection units difficult. Onsite installation, wiring, and long term maintenance further increase the difficulty of practical application, making it difficult to meet the online detection requirements of high speed knitting.

Condition monitoring using the actuator response signals can reduce reliance on external detection devices. In studies of piezoelectric needle selectors, the combined actuation and sensing capability of piezoelectric elements has been used for condition monitoring. Peng et al. [8] determined the operating condition of piezoelectric needle selectors by analyzing changes in piezoelectric actuation and sensing signals. Dai et al. [9] combined piezoelectric sensing signals with a closed loop control method to detect faults in a piezoelectric needle selection system. Ru et al. [10] further extracted condition features from the response signals of drive components for fault diagnosis of needle selector drive components. These studies show that response signals generated by the actuator itself can reflect information on its operating condition, providing a useful reference for condition monitoring without additional sensing devices [11,12]. Yet piezoelectric needle selectors and magnetic latching needle selectors differ in actuation mode, structural configuration, and energy conversion mechanism, making these detection methods difficult to apply to magnetic latching needle selectors.

Drive current is an internal electrical response during actuator operation and has been widely used for condition monitoring of electromechanical equipment [13,14]. Niu et al. [15] reviewed motor current signature analysis, showing how current reflects changes in equipment condition. Yakhni et al. [16] highlighted the value of transient currents for detecting faults in variable speed induction motors, while Diversi et al. [17] used autoregressive current analysis to capture fault-related temporal patterns. For electromagnetic actuators, Rahman et al. [18] linked solenoid current to armature position, Nagai et al. [19] estimated position and force from current variations, and König et al. [20] used eddy current effects to improve position estimation under dynamic conditions. In solenoid valves, drive current has also been used for fault detection and performance analysis. Liu et al. [21] identified spool abnormalities from current waveforms, while Ma et al. [22] improved fault discrimination through multi-feature fusion. More recently, Yoo et al. [23] diagnosed hydraulic solenoid valve faults using measured voltage and current, and Zhang et al. [24] combined simulated coil currents with experimental validation for circuit breaker fault diagnosis. These studies show that drive current contains information related to changes in actuator condition and can provide an important basis for equipment condition monitoring [25,26,27,28]. Beyond current-based feature analysis, dynamic system models have also been used for fault detection. Li et al. [29] developed finite-horizon fault detection filters for linear discrete time-varying systems with multiplicative noise, using system dynamics to construct fault-sensitive detection measures. Despite advances in signal-based and model-based fault detection, the short-pulse current response of magnetic latching needle selectors and its relationship with fault-related physical changes remain insufficiently understood. Identifying multiple states from these responses within the timing and computational constraints of circular knitting machine controllers remains a challenge [30,31].

This study focuses on the condition information contained in the transient drive current of magnetic latching needle selectors, investigates current responses under different fault conditions, and develops a lightweight identification method. An electromechanical coupling model is used to analyze the effects of initial swing angle, geometric offset, equivalent coil resistance, and core saturation magnetization through single-factor perturbations, examining changes in initial position, magnetic circuit geometry, coil resistance, and core magnetization. Within the 160 μs pulse, the drive current exhibits rapid initial growth, a slower intermediate increase, and terminal slope recovery, consistent with changes in core magnetization and dynamic inductance. Variations in these parameters mainly affect stage durations, growth trends, and current levels at key positions. Based on these differences, a 14-dimensional feature vector comprising eight-stage macro, three-stage dynamic, and three equivalent electromagnetic response features is constructed from measured currents, and a Gated Low-Rank Cross Network (GLR-CrossNet) is used to identify the normal state and five fault states. The method is deployed on the needle selector control terminal and evaluated through continuous operation tests on a circular knitting machine. The contribution lies in physically guided three-stage feature construction, compact fault identification, and validation on the needle selector control terminal. The paper is organized as follows: Section 2 analyzes the needle selector actuation mechanism and transient drive current response. Section 3 describes the fault specimens and drive current experiments. Section 4 presents the multistage condition features and GLR-CrossNet identification method. Section 5 analyzes the six condition identification results and presents comparative and ablation analyses, controller deployment, and continuous operation validation on the circular weft knitting machine. Section 6 concludes the paper.

## 2. Actuation Mechanism and Transient Current Response of Magnetic Latching Needle Selectors

### 2.1. Structure and Electromechanical Coupling Model of Magnetic Latching Needle Selectors

**Figure 1:**
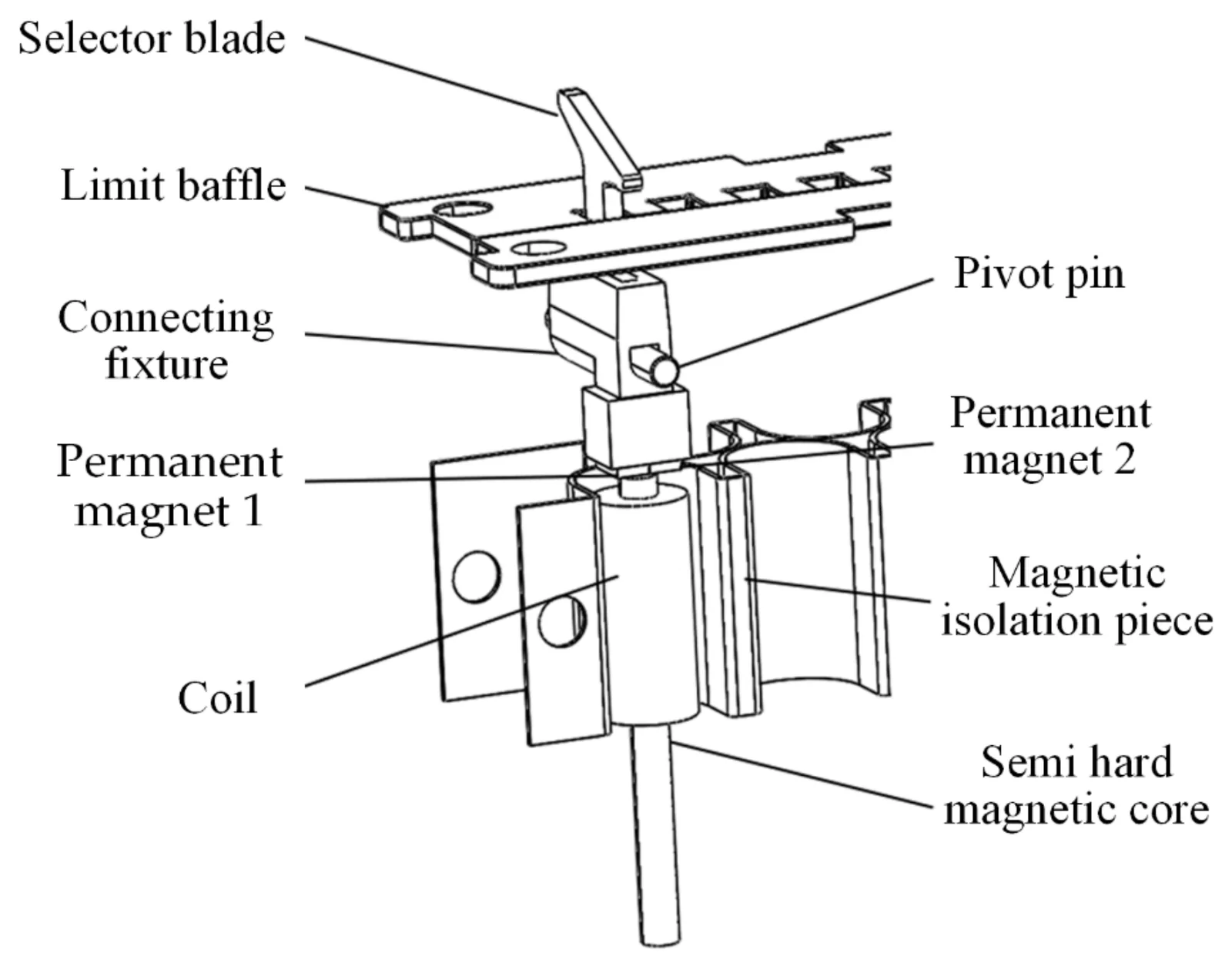
Structure of the magnetic latching needle selector.

As shown in Figure 1, the magnetic latching needle selector mainly consists of a selector blade, limit baffles, a permanent magnet assembly, a coil, a pivot pin, a magnetic isolation piece, and a semi-hard magnetic core. The permanent magnet assembly consists of two oppositely arranged permanent magnets and a connecting fixture. The assembly is connected to the selector blade, allowing the blade to swing about the pivot pin. The coil is wound around the semi-hard magnetic core, and the drive pulse changes the magnetization state of the core. The resulting magnetic field interacts with the bias field of the permanent magnets, producing electromagnetic torque that drives the selector blade toward the opposite limit position. After the pulse ends, the semi-hard magnetic core retains a certain magnetization state, and the magnetic interaction with the permanent magnets maintains the selector blade at the corresponding limit position.

The magnetization process of the needle selector is jointly determined by the coil circuit and nonlinear magnetic field. The coil terminal voltage satisfies:

(1) $$u(t)=R_{c}i(t)+\frac{d\lambda (t)}{dt}$$

where u(t), i(t), $R_{c}$ and λ(t) represent the drive voltage, coil current, equivalent coil resistance, and flux linkage, respectively. For an N turn coil, the flux linkage and the magnetic flux linked with the coil satisfy:

(2) $$\lambda (t)=N\Phi (t)=N\int_{A}B(t)\cdot dA$$

where Φ represents the magnetic flux linked with the coil and B represents the magnetic flux density. The magnetic flux density of the semi-hard magnetic core is expressed as:

(3) $$B(t)=\mu_{0}[H(t)+M(t)]$$

where H represents the magnetic field strength and M represents the magnetization.

Semi-hard magnetic materials exhibit hysteresis and remanence, and their magnetization state depends on the current excitation and previous magnetization process. The bias magnetic field generated by the permanent magnets also affects the magnetic field distribution in the core and air gap, while changes in the selector blade position alter the relative relationship between the permanent magnets and the core. Therefore, different initial angular positions correspond to different initial magnetic circuit states.

Nonlinear magnetization causes the coil inductance to vary with the operating point of the core. Under a given magnetization branch and geometric position, the dynamic inductance can be defined as:

(4) $$L_{\mathrm{dyn}}=\frac{d\lambda }{di}$$

Previous studies on electromagnetic actuators have also reported the sensitivity of inductance to magnetic field conditions and motion positions [19]. During the short term magnetization process, the change in selector blade position is relatively limited, and the coil terminal relationship can be expressed as:

(5) $$u(t)=R_{c}i(t)+L_{\mathrm{dyn}}(t)\cdot \frac{di(t)}{dt}$$

As the operating point of the core moves along the magnetization curve, $L_{\mathrm{dyn}}$ changes accordingly, and the growth rate of the drive current also varies. This relationship provides the basis for subsequent analysis of stage variations in the magnetization current.

Integrating Equation (1) over the pulse duration gives the change in flux linkage:

(6) $$\lambda (t)-\lambda (0)=\int_{0}^{t}[u(\tau )-R_{c}i(\tau )]d\tau$$

The interaction between the air gap magnetic field and the permanent magnets generates electromagnetic torque. For the complete reversal process, the selector blade and permanent magnet assembly can be approximated as a single degree of freedom system rotating about the pivot pin. Its dynamic relationship is:

(7) $$J\cdot \frac{d\omega }{dt}=T_{e}-T_{f}-T_{\lim}$$

The angular velocity and angular displacement of the selector blade satisfy:

(8) $$\frac{d\theta }{dt}=\omega$$

where J, $T_{e}$, $T_{f}$, and $T_{\lim}$ denote the equivalent rotational inertia of the moving assembly, electromagnetic torque, resistance torque due to friction and damping, and constraint torque arising from limit contact, respectively.

**Figure 2:**
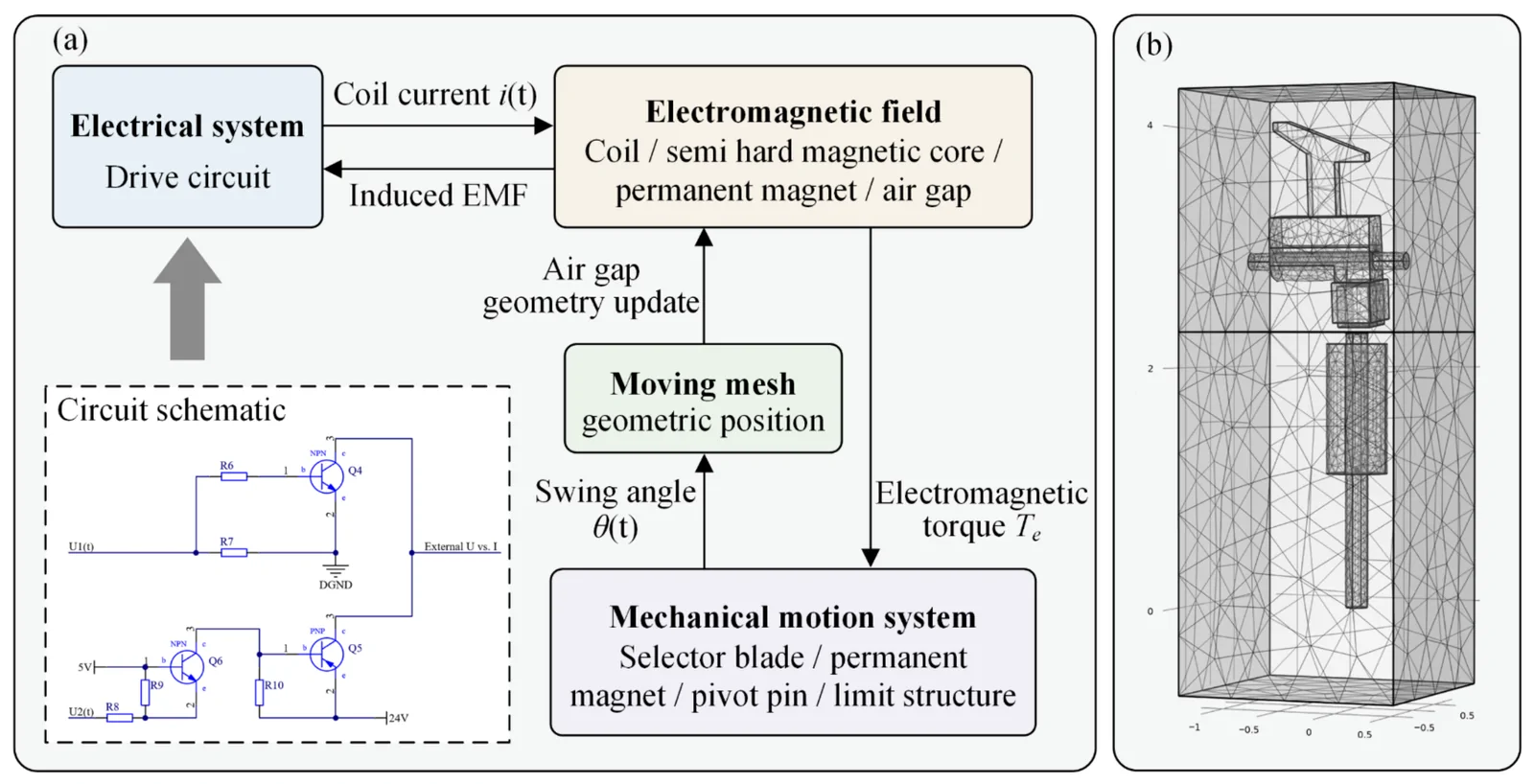
Multiphysics model of the magnetic latching needle selector: (**a**) Coupling relationship among electrical, electromagnetic, and mechanical domains. (**b**) Finite element model.

Based on the above electromechanical relationships, the multiphysics model shown in Figure 2 is established. The geometric model is constructed according to the actual needle selector structure, including the coil, semi-hard magnetic core, permanent magnets, air domain, and moving domain, with the pivot pin defined as the rotation center of the selector blade assembly. Numerical calculations use the Jiles–Atherton model to describe the hysteresis characteristics of the semi-hard magnetic core [32]. The model parameters are $M_{s}=1.9\times 10^{6}\;A/m$ (saturation magnetization), a=5500 A/m (anhysteretic curve shape parameter), k=25,000 A/m (pinning parameter), c=0.03 (reversibility coefficient), and $\alpha =4\times 10^{-5}$ (mean-field coupling parameter). The air gap electromagnetic torque is calculated using Maxwell stress [33]. Under the reference condition, the drive voltage is 24 V, the pulse width is 160 μs, and the time step for transient calculation is 1 μs. The model simultaneously outputs the drive current, core magnetization state, magnetic flux density, electromagnetic torque, and selector blade angular displacement.

### 2.2. Three-Stage Transient Current Response and Stage Features Under the Reference State

**Figure 3:**
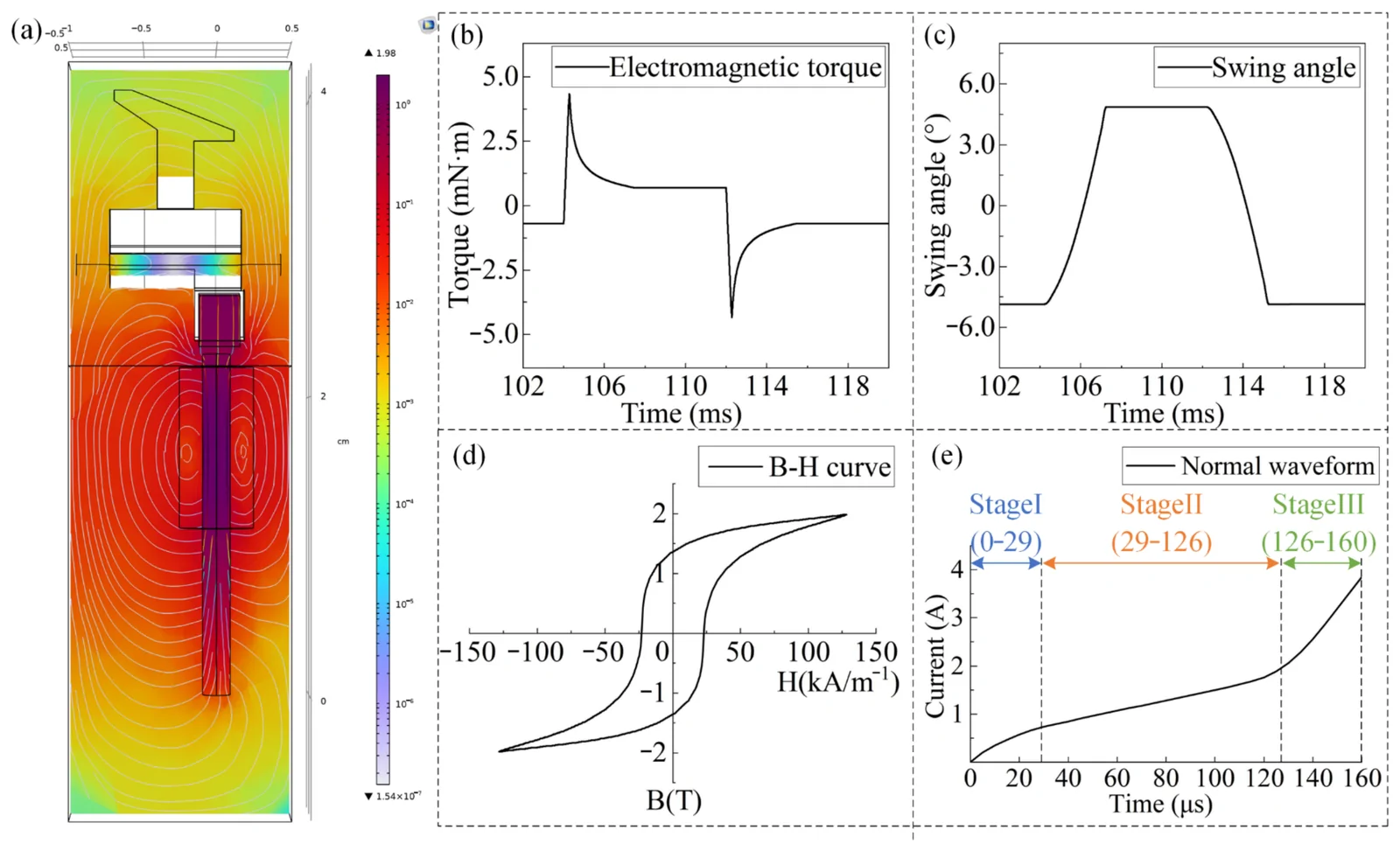
Electromagnetic, mechanical, and drive current responses of the magnetic latching needle selector under the normal state: (**a**) Magnetic flux density and magnetic field distribution. (**b**) Electromagnetic torque response. (**c**) Selector blade swing angle response. (**d**) B–H curve of the semi-hard magnetic core. (**e**) Three-stage transient drive current response.

Figure 3 shows the electromagnetic, mechanical, and drive current responses of the magnetic latching needle selector under the reference condition. After pulse excitation, the magnetic flux mainly forms a closed magnetic circuit through the semi-hard magnetic core, working air gap, and permanent magnet assembly. The magnetic flux density and magnetic field distribution at the end of magnetization are shown in Figure 3a. Figure 3b and c show the electromagnetic torque and selector blade angular displacement responses, respectively. Figure 3d presents the simulated B–H hysteresis loop obtained using the Jiles–Atherton model, with a remanent flux density $B_{r}$ of approximately 1.36 T and a coercive field $h_{c}$ of approximately 23.12 kA/m. The corresponding drive current is shown in Figure 3e. The current continuously increases within 160 μs and exhibits two clear slope transitions.

At the beginning of the pulse, the current rapidly increases, and the core moves from the initial operating state into the main magnetization region. As the operating point moves along the nonlinear magnetization curve, the dynamic inductance changes and the current slope gradually decreases. This interval is defined as stage I. The current then enters a relatively long slow increasing region, corresponding to stage II. In the later part of the pulse, as the core approaches the high-field region, the local magnetization increment and dynamic inductance predicted by the model decrease, allowing the current slope to increase again. This interval is defined as stage III. In the reference waveform shown in Figure 3e, the stage boundaries occur at approximately 29 and 126 μs.

The main differences among the three current stages are reflected in stage duration, growth trend, and current levels at key positions. Based on these variations, macro features are extracted from three aspects: time, slope, and amplitude. Let the boundary between stage I and stage II be denoted as $t_{b1}$, and the boundary between stage II and stage III be denoted as $t_{b2}$. The durations of stage I and stage II are defined as:

(9) $$\tau_{1}=t_{b1}$$

(10) $$\tau_{2}=t_{b2}-t_{b1}$$

Since the total current pulse width is 160 μs, the duration of stage III can be determined from the boundaries of the first two stages and is therefore not independently used as a feature. The currents within the three stages exhibit relatively stable overall increasing trends. To reduce the influence of local fluctuations on slope estimation, each stage current is fitted using first-order Huber regression:

(11) $$i(t)=k_{j}t+b_{j},t\in \Omega_{j},j=1,2,3$$

where *j*th denotes the stage index, $k_{1}$, $k_{2}$, and $k_{3}$ are the trend slopes of the three stages, and $b_{j}$ is the corresponding fitting intercept. To retain the current levels at the stage boundaries and pulse end, the following features are defined:

(12) $$I_{1}=i(t){|}_{t=t_{b1}}$$

(13) $$I_{2}=i(t){|}_{t=t_{b2}}$$

(14) $$I_{3}=i(t){|}_{t=T_{p}},T_{p}=160\;\mathrm{\mu s}$$

The final eight-dimensional stage macro feature vector is obtained:

(15) $$x_{mac}={\left[\tau_{1},\tau_{2},k_{1},k_{2},k_{3},I_{1},I_{2},I_{3}\right]}^{T}$$

where $\tau_{1}$ and $\tau_{2}$ describe the time variations of different stages, $k_{1}$ to $k_{3}$ represent the current growth characteristics of the three stages, and $I_{1}$ to $I_{3}$ retain the current levels at the stage boundaries and pulse end. These three types of features correspond to the differences in waveform time position, variation trend, and amplitude, and can characterize the transient drive current responses caused by different parameter variations.

### 2.3. Effects of Four Parameter Perturbations on Drive Current and Selector Blade Motion

Based on the above electromechanical coupling relationships, the drive current variation during the 160 μs magnetization process is closely related to the initial magnetic circuit state, relative positions of magnetic components, coil resistance, and core magnetization characteristics. The initial swing angle $\theta_{0}$, geometric offset Δx, equivalent coil resistance $R_{c}$, and core saturation magnetization $M_{s}$ were selected to represent these four influences, respectively. Each parameter was varied independently, with the others held at their reference values.

**Figure 4:**
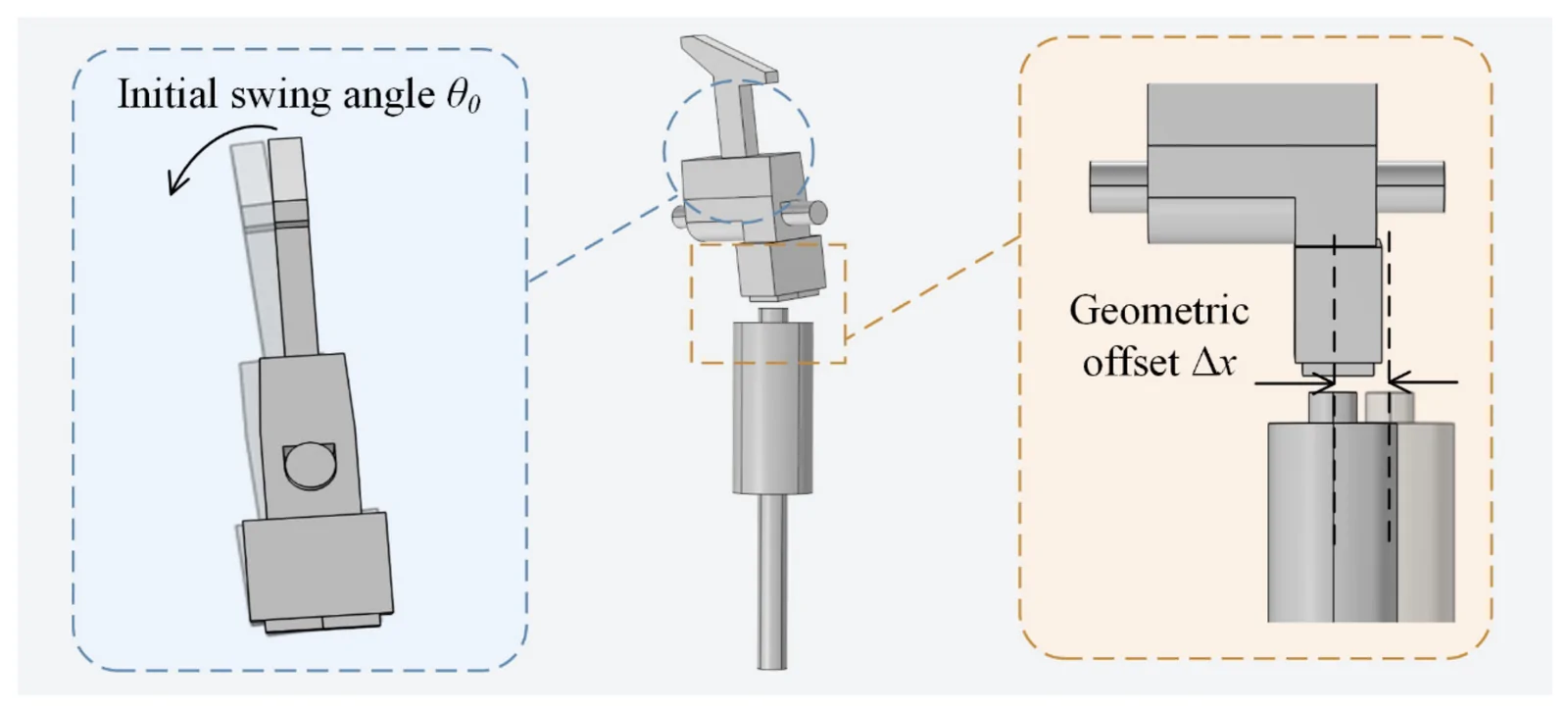
Schematic of the initial swing angle and geometric offset.

Here, $\theta_{0}$ denotes the initial swing angle of the selector blade before pulse excitation, while Δx describes the relative geometric offset between key magnetic components. Their definitions are shown in Figure 4. The specific perturbation ranges of the four parameters are listed in Table 1.

**Figure 5:**
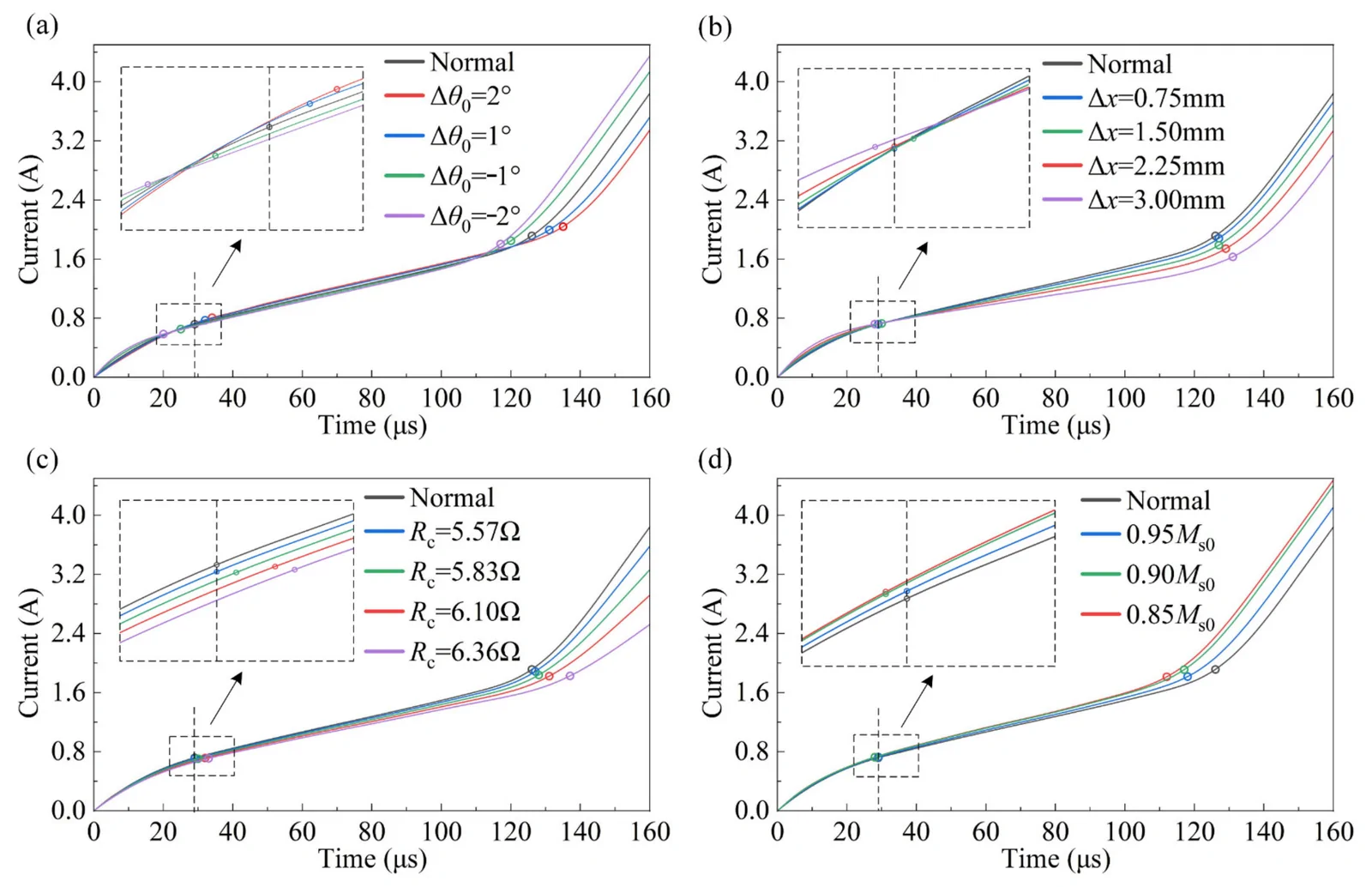
Drive current responses to four parameter perturbations: (**a**) Initial swing angle $\theta_{0}$. (**b**) Geometric offset Δx. (**c**) Equivalent coil resistance $R_{c}$. (**d**) Saturation magnetization $M_{s}$.

The drive currents under different parameter conditions are shown in Figure 5. After varying $\theta_{0}$, waveform differences appear earlier, and both the growth trend of stage I and the stage transition positions change throughout the subsequent magnetization process. This is because $\theta_{0}$ changes the magnetic circuit state among the permanent magnets, core, and working air gap. The influence of Δx on the early current response is relatively weak. As the magnetization process proceeds, the curves gradually separate in stage II, and the slope and terminal current level in stage III also change. Although $\theta_{0}$ and Δx both modify the magnetic circuit conditions, their effects are most evident in different stages of the current response.

After increasing $R_{c}$, the differences among stage I curves are relatively small, while the current levels become progressively lower than those under the reference condition in stages II and III, with the most pronounced differences in stage III. As the coil current increases, the voltage drop across $R_{c}$ increases, reducing the voltage component available for flux linkage variation. Changes in $M_{s}$ modify the magnetization process of the semi-hard magnetic core, resulting in variations in the current trends of stages II and III. Under some perturbation levels, increased current amplitude does not lead to faster selector blade response, producing different response characteristics from simply increasing $R_{c}$.

**Figure 6:**
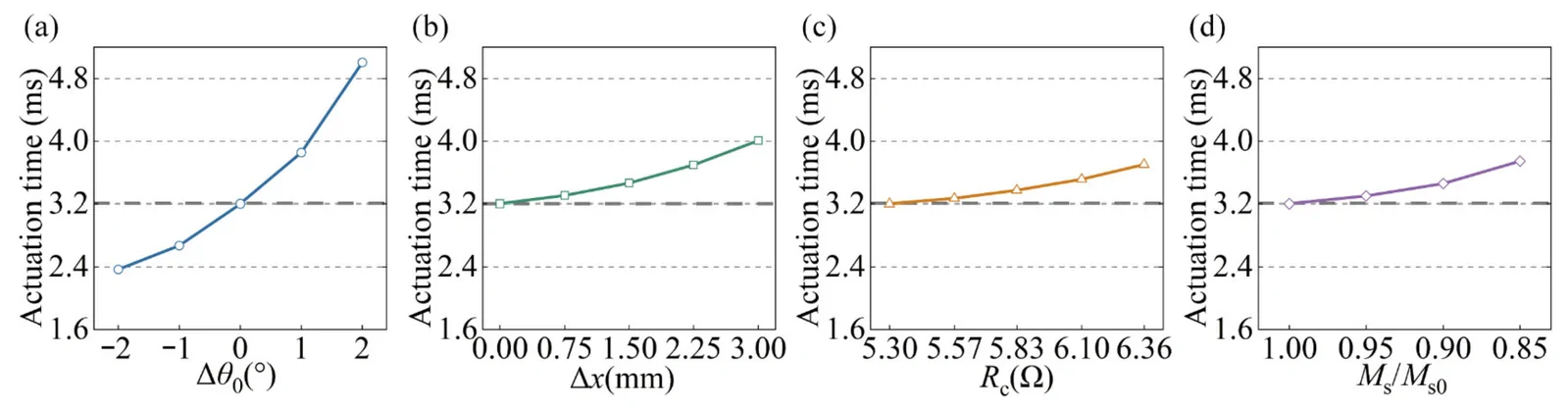
Selector blade actuation time under four parameter perturbations: (**a**) Initial swing angle $\theta_{0}$. (**b**) Geometric offset Δx. (**c**) Equivalent coil resistance $R_{c}$. (**d**) Saturation magnetization $M_{s}$.

These differences can also be observed in the selector blade actuation time, as shown in Figure 6. Variations in $\theta_{0}$ simultaneously change the initial magnetic circuit condition and the rotation angle required for selector blade reversal, resulting in significant changes in actuation time. With increasing Δx, the effective magnetic field and electromagnetic torque are affected, leading to changes in selector blade actuation time. Increasing $R_{c}$ suppresses the magnetization current and gradually prolongs the actuation process. After reducing $M_{s}$, the selector blade response becomes slower even when the terminal current increases during some stages. Therefore, similar current amplitude variations may correspond to different electromagnetic and mechanical states, and a single amplitude feature is insufficient to distinguish different parameter variations.

**Figure 7:**
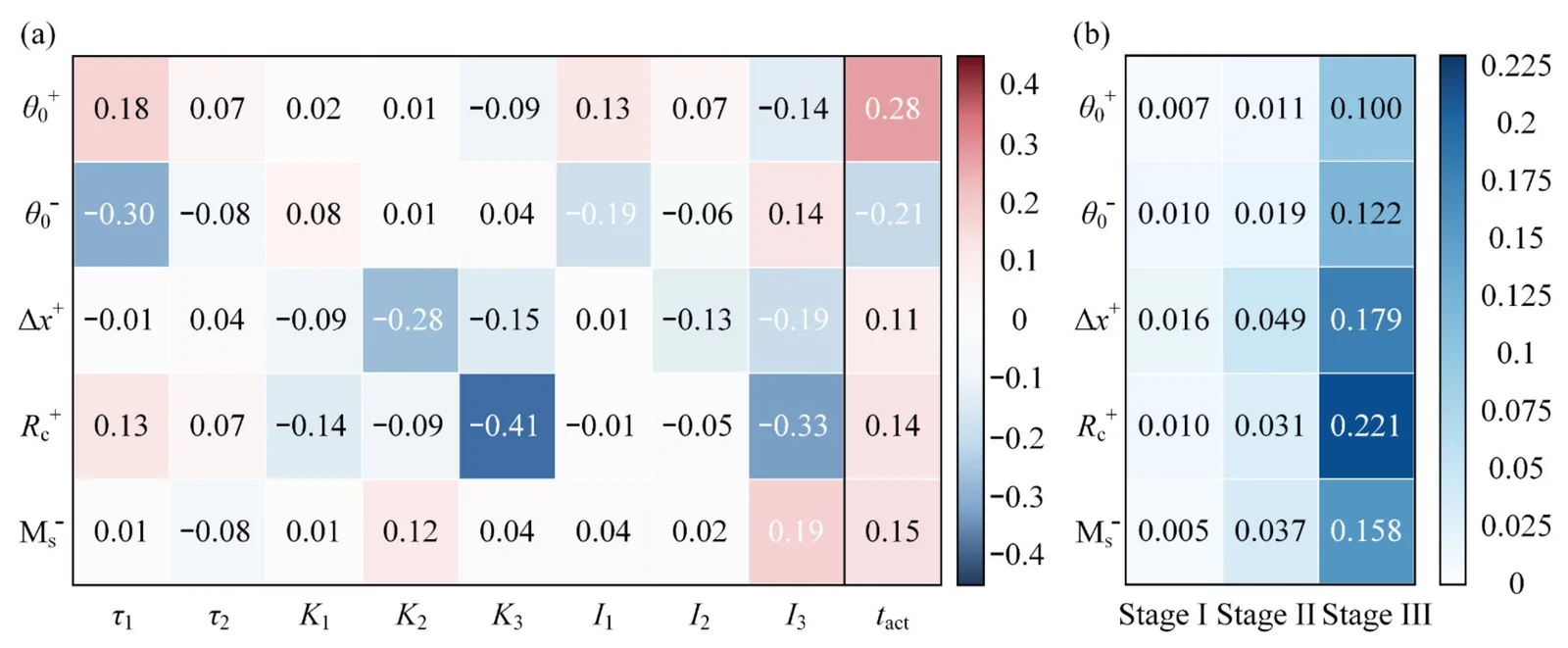
Normalized directional sensitivities of stage macro features and selector blade actuation time under four parameter perturbations: (**a**) Normalized directional sensitivity of each feature and actuation time. (**b**) Stagewise waveform deviation under the maximum perturbation level.

Figure 7 further summarizes the normalized directional sensitivity of macro features and selector blade actuation time within the specified perturbation ranges. Variations in $\theta_{0}$ mainly affect stage duration and the early stage slope. The influence of Δx is more significant on stages II and III. Increasing $R_{c}$ mainly changes the middle and later stage slopes and the terminal current level. A reduction in $M_{s}$ simultaneously affects the later stage trend and key current levels, while sensitive features corresponding to different parameters are not concentrated in the same stage.

Therefore, the influence of the four parameters on drive current is reflected not only in amplitude variation but also in changes in stage position and growth trend. The eight stage macro features capture these variations and, together with stage dynamic and equivalent electromagnetic response features, characterize differences in measured drive currents among the six states.

## 3. Fault Specimens and Drive Current Experiments

### 3.1. Drive Current and Selector Blade Motion Measurement Methods

**Figure 8:**
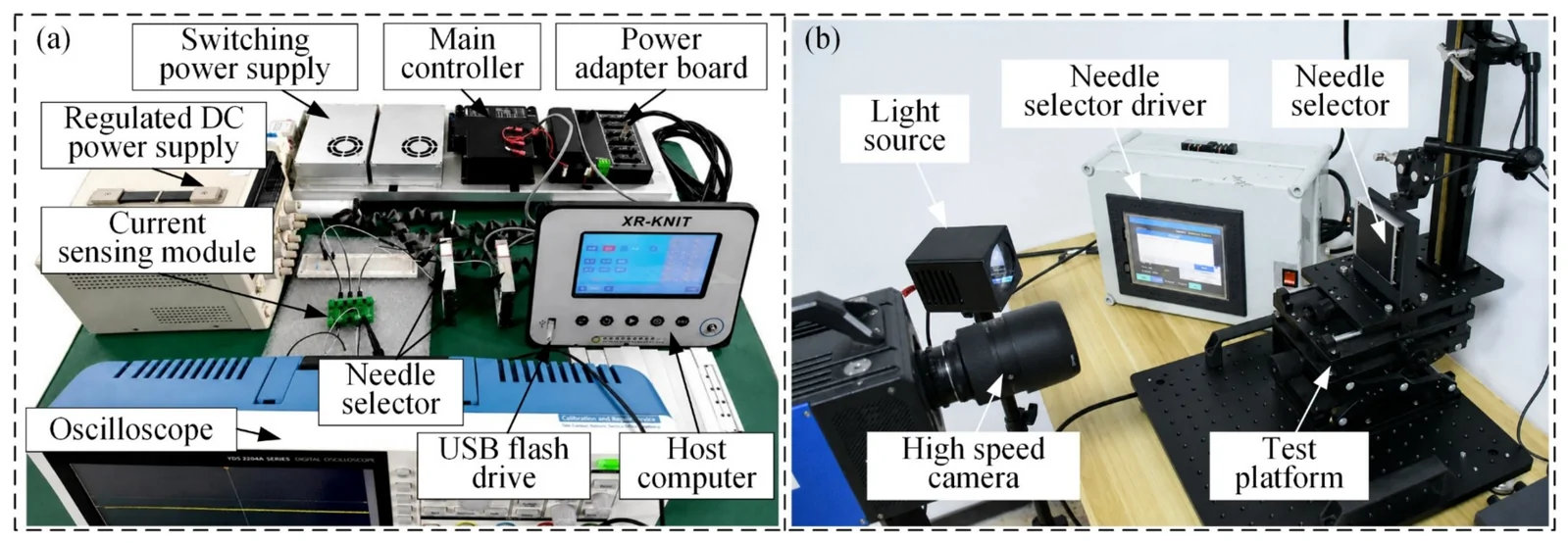
Experimental platforms for drive current and selector blade motion measurement: (**a**) Drive current acquisition platform. (**b**) High speed vision testing platform for selector blade motion.

To measure the transient drive current and selector blade motion during needle selector operation, a drive current acquisition platform and a high-speed vision testing platform were established, as shown in Figure 8.

Two acquisition methods were used for drive current measurement. The high-resolution reference waveform was acquired using an oscilloscope at a sampling rate of 3.125 MS/s, obtaining approximately 500 data points within the 160 μs pulse. These data were used to observe stage transitions and local waveform variations and to compare with the normal-state simulation. During actual control, the microcontroller at the needle selector side samples the current at 1 μs intervals, obtaining a 161-point sequence within the range of 0 to 160 μs for each action. The acquired sequences are transmitted to the main controller via CAN and saved to a USB flash drive through the host computer to construct the dataset for model training and testing. Online identification uses the same current sampling scheme on the control terminal.

The high-speed vision system records selector blade motion. Blade displacement and actuation time are extracted from successive image frames to characterize the mechanical response under different conditions.

**Figure 9:**
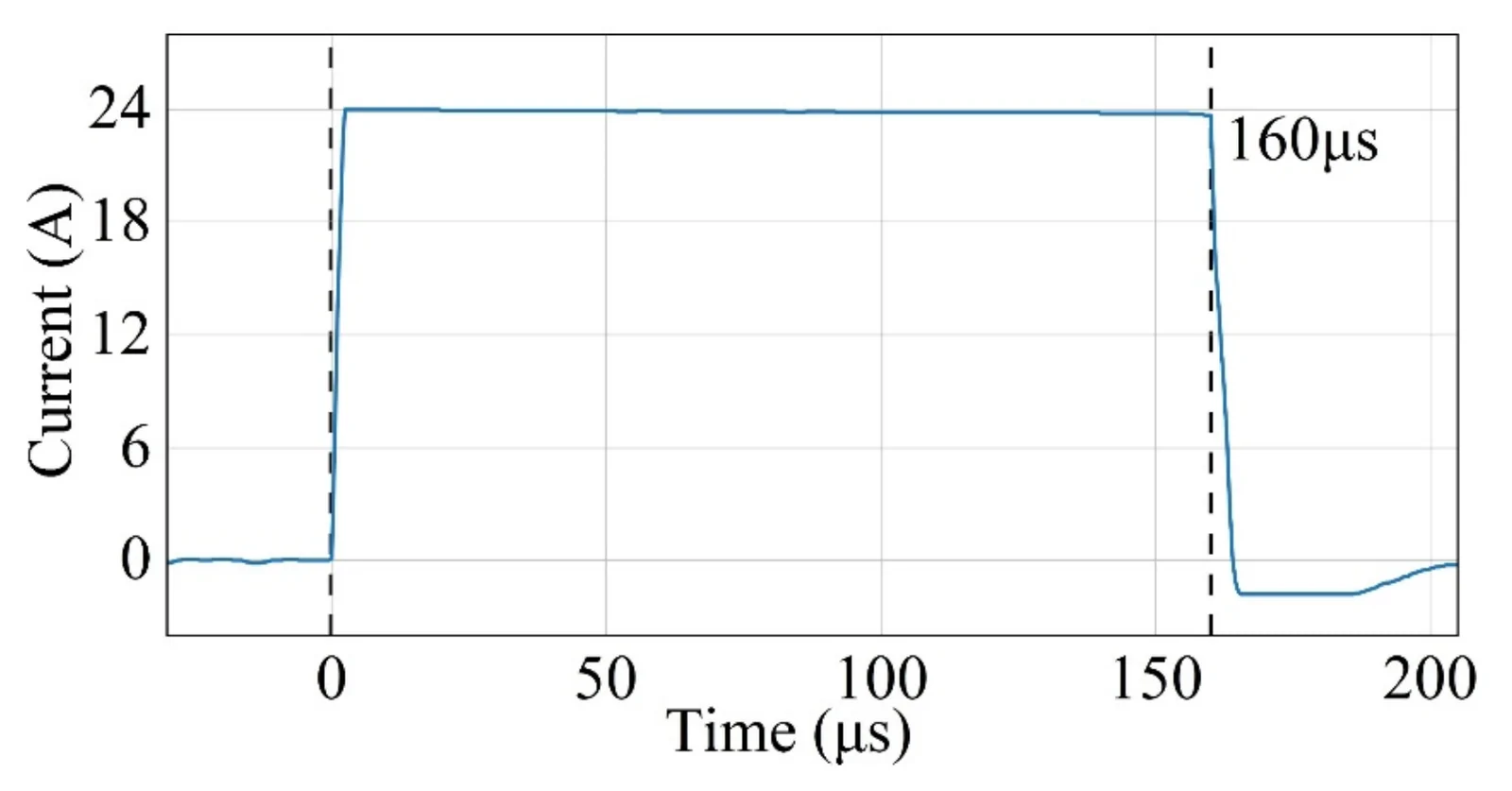
Measured coil-terminal voltage waveform during the 160 μs excitation pulse.

In both measurement setups, the needle selector was driven by a nominal 24 V DC pulse with a width of 160 μs. The measured coil-terminal voltage was approximately 23.7–24 V during the pulse plateau, as shown in Figure 9.

### 3.2. Comparison Between Normal State Response and Simulation Results

**Figure 10:**
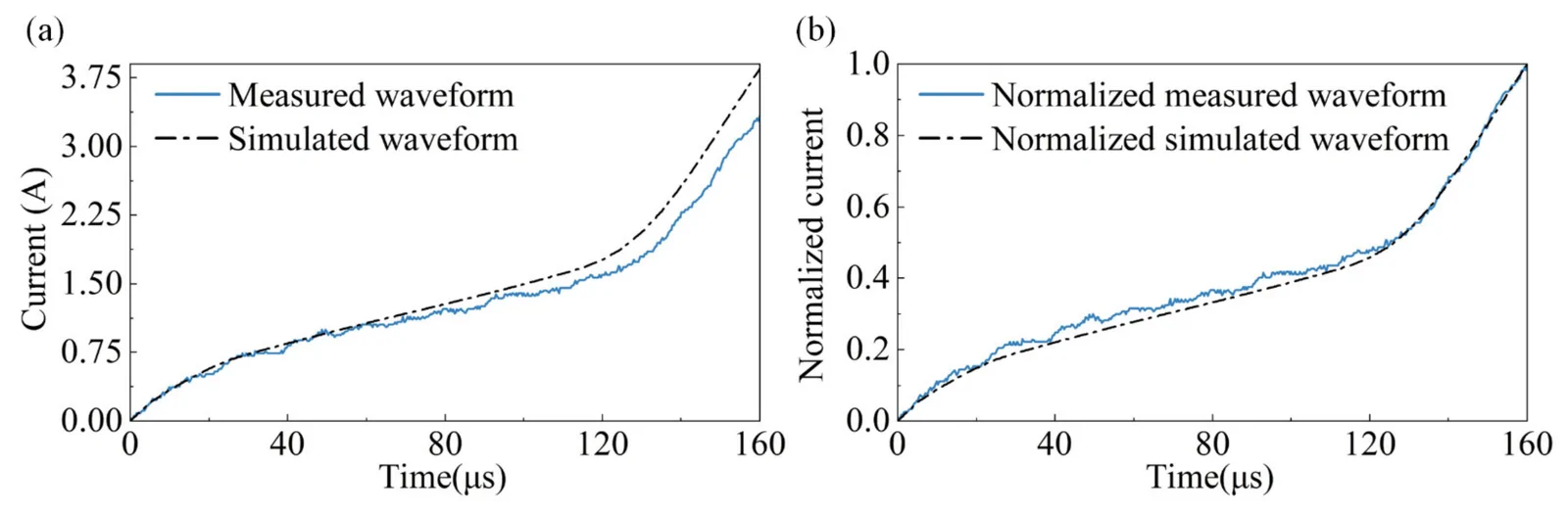
Comparison between simulated and measured drive currents under the normal state: (**a**) Simulated and measured drive currents. (**b**) Normalized simulated and measured drive currents.

Normal specimens with complete structures and stable actions were selected for repeated tests. The representative measured drive current and simulated drive current obtained from the reference model are shown in Figure 10a. Both curves exhibit rapid initial growth, slower intermediate growth, and terminal slope recovery within the 160 μs pulse, with similar positions of the main slope transitions. Although the measured waveform contains certain local fluctuations and amplitude deviations, the three-stage response remains clear.

Figure 10b shows the two currents normalized by their respective peak values, highlighting similarities in waveform shape and stage transitions. Based on 500 paired samples over 0–160 μs, the root mean square error (RMSE) was 0.1921 A, and the RMSE normalized by the measured peak current (NRMSE) was 5.76%. The simulated peak exceeded the measured peak by approximately 0.51 A, indicating some amplitude overestimation near the end of the pulse. The reference model thus captures the main growth trend and three-stage characteristics of the normal-state current.

**Figure 11:**
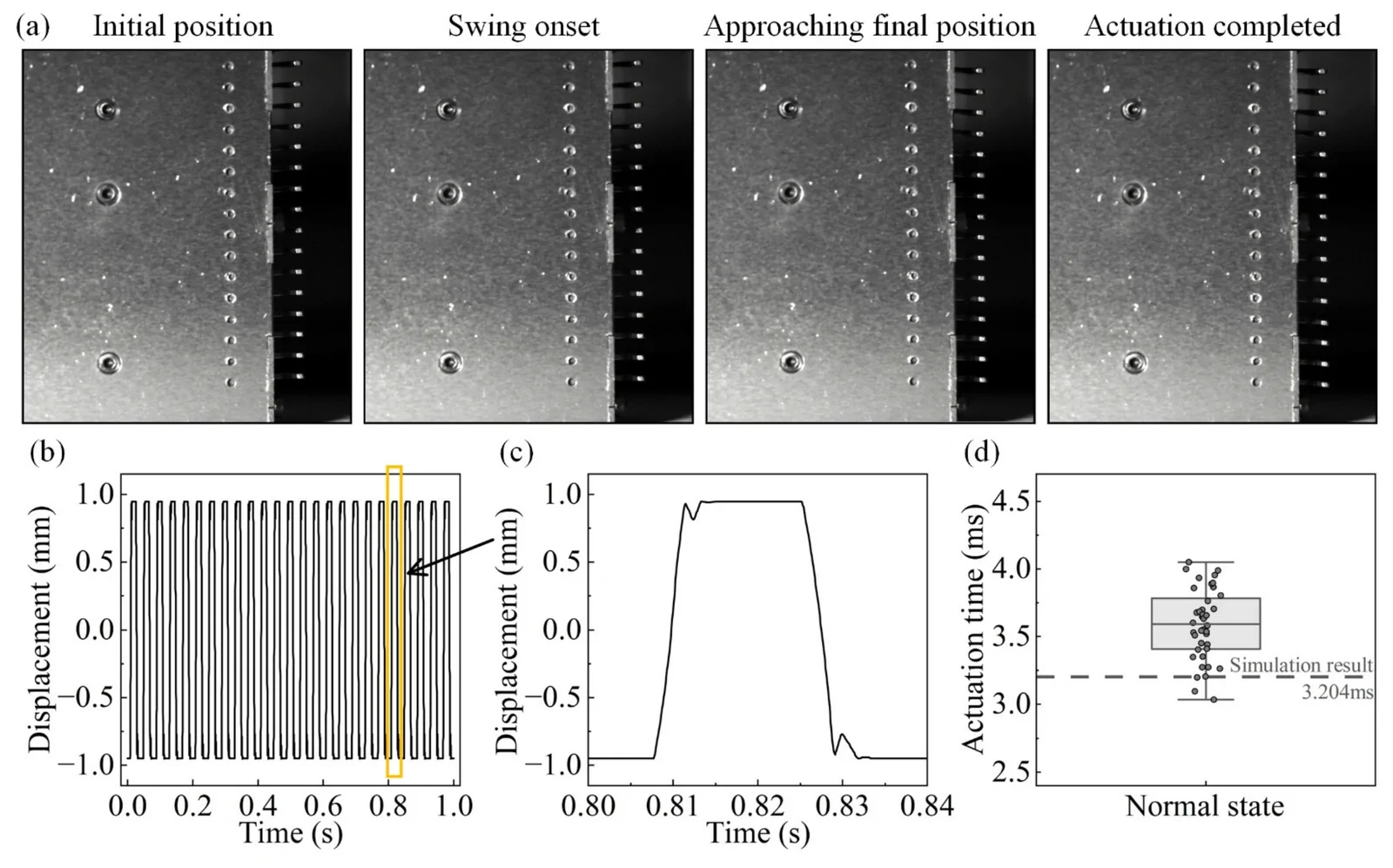
Selector blade motion and visual displacement extraction results under the normal state: (**a**) Sequential images of the selector blade actuation process. (**b**) Target interval extraction from the displacement sequence. (**c**) Displacement response of a single actuation. (**d**) Actuation time distribution and simulation result.

The mechanical motion of the normal specimen was verified using high-speed vision images, and the results are shown in Figure 11. Figure 11a records the complete process of the selector blade starting from the initial holding position, swinging, and reaching the target position. Figure 11b shows the continuously recorded blade displacement and the selected actuation interval, while Figure 11c presents the displacement response within this interval after coordinate-to-displacement conversion. In the established model, the selector blade completes the swinging motion within 3.204 ms. Figure 11d compares the simulated actuation time with the measured distribution for normal specimens. The simulated value falls within the measured range, showing reasonable agreement with the experimental results.

### 3.3. Fault Specimens and State Determination

**Figure 12:**
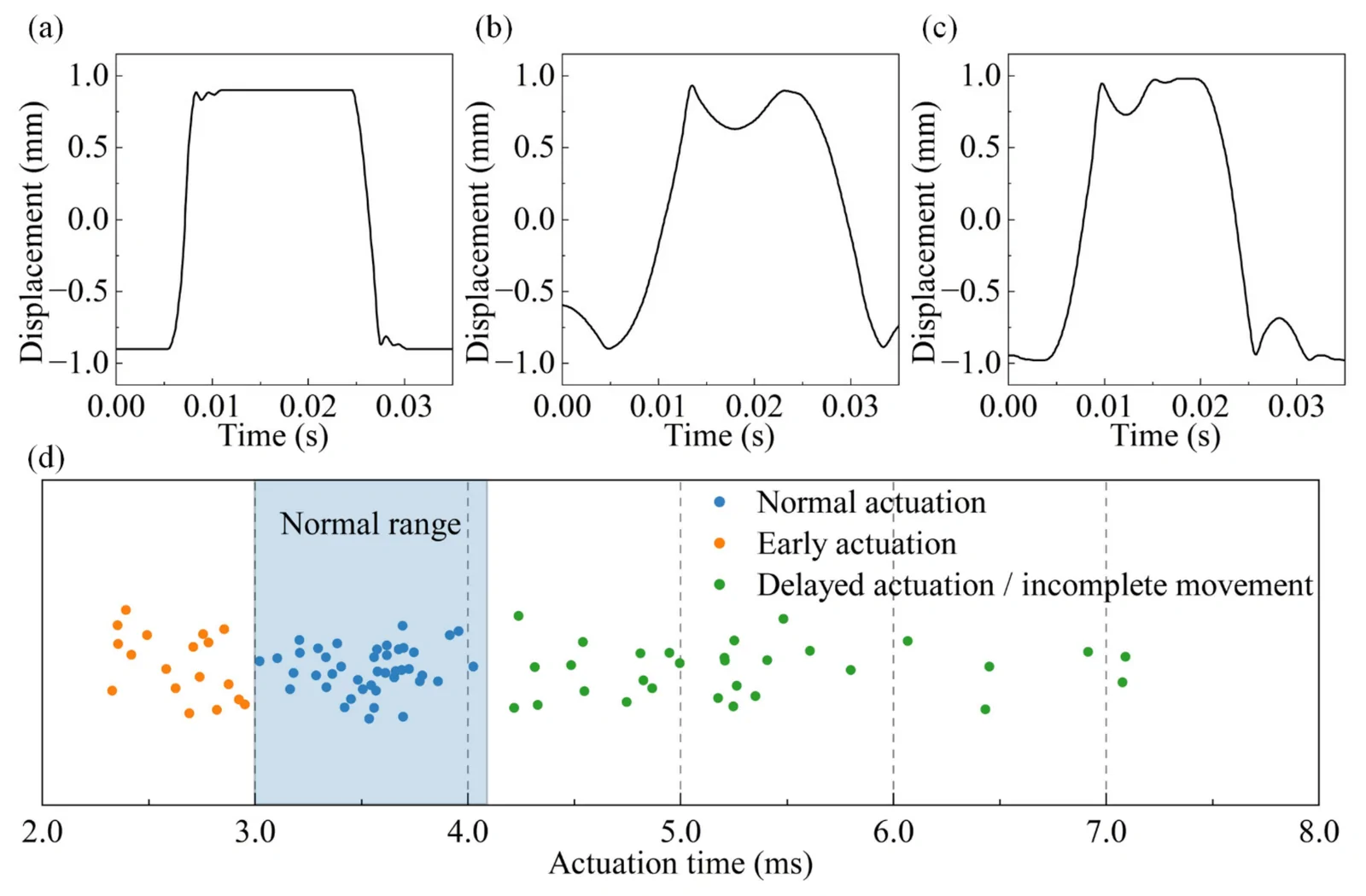
Typical selector blade motion responses of fault specimens: (**a**) Displacement response with early actuation. (**b**) Displacement response with delayed actuation. (**c**) Displacement response with incomplete movement. (**d**) Actuation time distribution.

During high-speed vision testing, abnormal specimens mainly exhibited three types of mechanical responses: early actuation, delayed actuation, and incomplete movement. Typical results are shown in Figure 12. As similar motion responses may occur in different states, specimens were further classified through structural inspection, coil resistance measurements, and core magnetic response tests.

**Figure 13:**
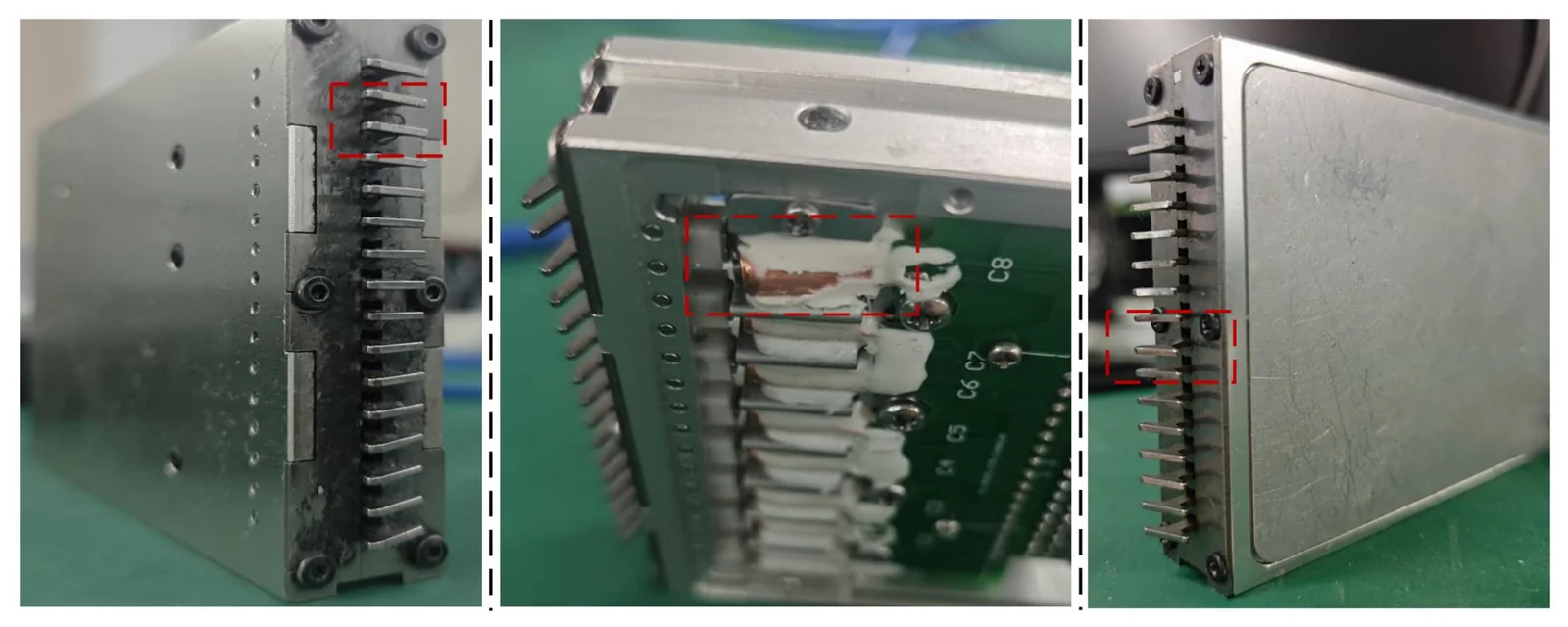
Typical appearance states of DB, CM, and BW fault specimens.

Figure 13 shows representative specimens exhibiting debris blockage (DB), coil misalignment (CM), and baffle wear (BW). In DB specimens, debris accumulation in the return region shifts the stable holding position of the selector blade, altering its initial swing angle and causing early actuation. CM specimens exhibit apparent coil expansion or position misalignment. This changes the relative positions of the coil, core, and permanent magnet assembly, preventing the blade from reaching its normal final position. In BW specimens, repeated impact causes wear and position deviation at the limit contact surface, altering the initial position and reversal stroke and prolonging actuation time.

Coil degradation (CD) is usually not apparent from visual inspection. Coil resistance was measured at room temperature. CD specimens showed higher resistance than normal specimens and delayed selector blade actuation.

**Figure 14:**
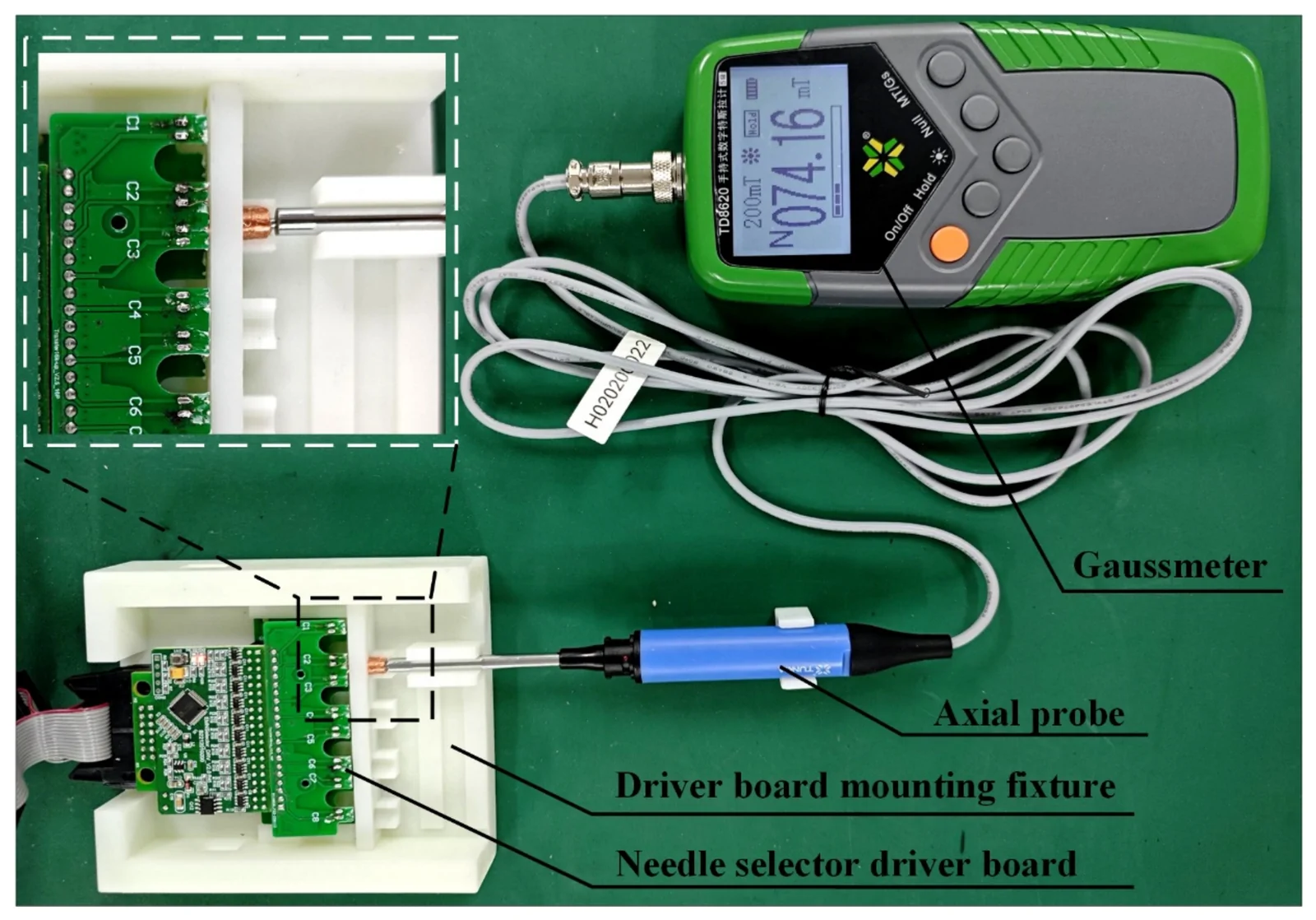
Magnetic response test setup for the semi-hard magnetic core and magnetic flux density measurement positions.

Core aging (CA) was characterized using the magnetic response test shown in Figure 14. Under the same excitation conditions and probe position, the peak axial magnetic flux density was 74.5 ± 4.7 mT for normal cores and 61.2 ± 6.8 mT for CA specimens. A more severely degraded specimen showed a minimum of 52.4 mT. Reduced magnetic response in CA specimens was also accompanied by delayed actuation.

Normal specimens (NM) showed stable and complete reversal, with intact structures, coil resistance, and core magnetic responses within normal ranges. Table 2 summarizes the six states and their determination criteria.

### 3.4. Measured Drive Current Features Under Six States

**Figure 15:**
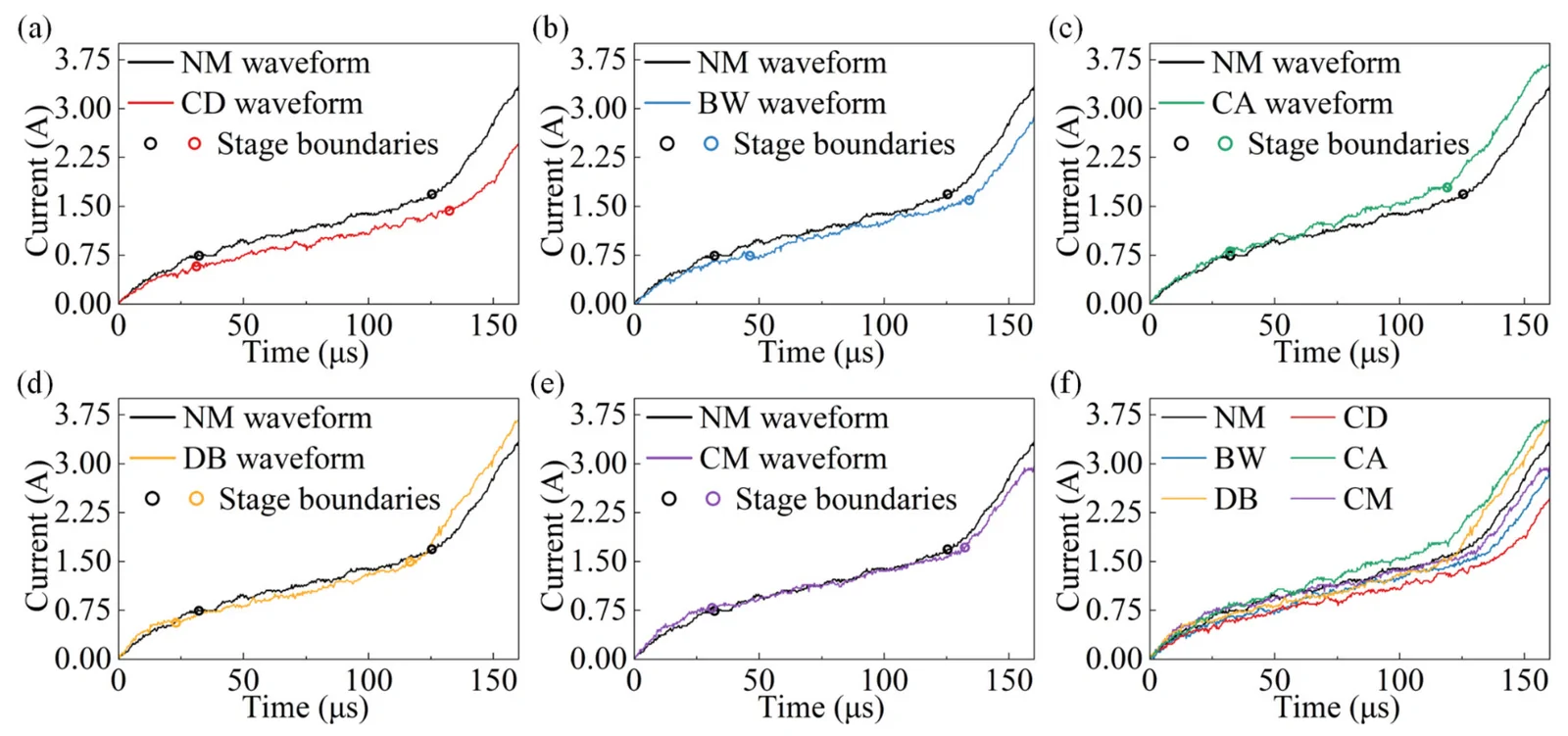
Representative measured drive currents under six states: (**a**) Comparison between CD and NM waveforms. (**b**) Comparison between BW and NM waveforms. (**c**) Comparison between CA and NM waveforms. (**d**) Comparison between DB and NM waveforms. (**e**) Comparison between CM and NM waveforms. (**f**) Waveforms under six states.

Representative measured drive currents of the six states are shown in Figure 15. Figure 15a–e compare CD, BW, CA, DB, and CM with NM, respectively, while Figure 15f shows the currents of all six states. All states retain the basic three-stage characteristics of the magnetization process, but differ in stage boundaries, growth rates, and current levels. Local spikes and fluctuations are also present in the measured waveforms.

Although CD, BW, and CA all exhibit delayed actuation of the selector blade, their current responses differ. The CD waveform is close to NM in the early pulse stage, while the difference gradually increases after entering stage II. In stage III, the growth rate and terminal current are both reduced. BW differs from NM in growth trend and transition position as early as stage I, with further current-level differences in stages II and III. CA exhibits faster current growth in stage II and higher current than NM over some intervals, despite its delayed actuation.

DB and CM exhibit distinct mechanical responses, and their waveform variations are concentrated in different regions. DB mainly affects stage I, changing its growth rate and the first transition position. The difference from NM then decreases, and the relative current amplitudes may reverse near the pulse end. CM remains close to NM initially but shows differences from stage II onward, including a changed stage III growth rate and lower terminal current. As shown in Figure 15f, the current waveforms of some fault states overlap over portions of the pulse, making a single time-point current insufficient to reliably distinguish all six states.

These differences involve stage duration, growth trend, current levels at key positions, and local variations within each stage. The subsequent feature construction therefore retains the stage macro features and further extracts within-stage dynamic information to capture local differences in the measured current waveforms.

## 4. Lightweight Fault Identification Method Based on Multi-Stage Transient Drive Current Response

### 4.1. Overall Identification Process

**Figure 16:**
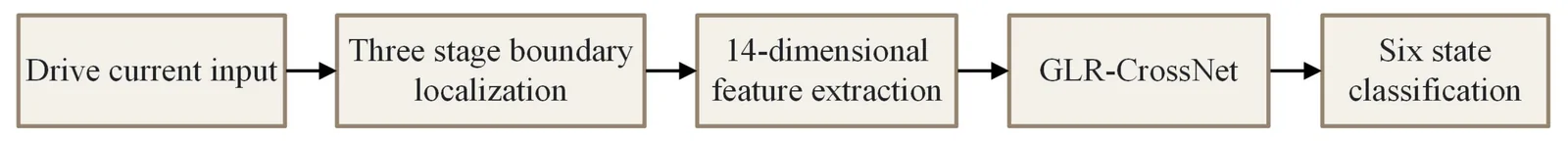
Six-state identification process based on multi-stage transient drive current responses.

The transient drive current generated during the operation of the magnetic latching needle selector contains response differences among different states. Based on the current variation analysis in Section 2 and the measured responses in Section 3, a six-state identification process is established, as shown in Figure 16.

The selector control terminal acquires the drive current during the 160 μs magnetization process and obtains a continuous 161-point current sequence. The sequence is divided into three response stages by locating two stage boundaries. Eight-stage macro, three-stage dynamic, and three equivalent electromagnetic response features are then extracted from the measured current to form a 14-dimensional feature vector. The Gated Low-Rank Cross Network (GLR-CrossNet) performs feature fusion and state discrimination through grouped gated recalibration and low-rank cross layers, outputting the identification results for the normal state and five fault states.

### 4.2. Construction of Multi-Stage Response Features

The measured drive current contains response differences among different states. However, direct feature extraction from the raw sequence may be affected by local fluctuations and random disturbances. Therefore, the current response is divided into different stages, and features related to state variations are extracted.

A three-point median filter is first applied to suppress isolated spikes, followed by a 13-point, third-order Savitzky–Golay (SG) polynomial fitting to obtain the current trend and local slope sequence. Based on the three-stage current response characteristics analyzed in Section 2, two stage boundaries $t_{b1}$ and $t_{b2}$ are determined according to the slope variations. For the j-th stage boundary, the median local slopes before and after the transition are calculated as $s_{\mathrm{pre}}$ and $s_{\mathrm{post}}$, respectively. The adaptive threshold is defined as:

(16) $$s_{\mathrm{th},j}=s_{\mathrm{pre},j}+\lambda_{j}(s_{\mathrm{post},j}-s_{\mathrm{pre},j}),j=1,2$$

where $\lambda_{j}$ is the threshold coefficient. In this study, the threshold coefficients for the first and second stage transitions are set to $\lambda_{1}$ = 0.50 and $\lambda_{2}$ = 0.23, respectively. For the first boundary, the pre- and post-transition reference intervals are 5–15 μs and 60–75 μs, with a search range of 18–52 μs; for the second, the corresponding intervals are 70–105 μs and 145–155 μs, with the search starting at 108 μs. The first boundary corresponds to a sustained decrease in local slope, whereas the second boundary represents a sustained slope recovery near the end of the pulse. During boundary searching, both the slope variation direction and continuity are considered. The first position satisfying the corresponding threshold condition for five consecutive samples is selected as the stage boundary.

After locating the two stage boundaries, the drive current is divided into three response stages. Stage duration, overall variation trend, and current values at key positions are extracted as stage macro features. The stage slopes are obtained using Huber linear regression:

(17) $$i(t)=k_{j}t+b_{j},j=1,2,3$$

where $k_{j}$ represents the average variation trend of stage j, and $b_{j}$ is the fitting intercept. Compared with ordinary least-squares fitting, Huber regression reduces the influence of abnormal samples on slope estimation. Eight stage macro features are constructed from the stage duration, slopes, and key current values.

In addition to the overall variation trend, local dynamic information within each stage also reflects state differences. Therefore, the current increment sequence is analyzed:

(18) $$\Delta i(n)=\tilde{i}(n+1)-\tilde{i}(n)$$

where $\tilde{i}(n)$ represents the processed current sequence. A first-order autoregressive (AR) model is then established:

(19) $$\Delta i(n)=c_{j}+a_{j}\Delta i(n-1)+\varepsilon (n)$$

where $a_{j}$ is the autoregressive coefficient of stage j, $c_{j}$ is the model intercept, and ε(n) is the residual term. The three autoregressive coefficients form the three-dimensional stage dynamic features describing local response variations. Autoregressive modeling has also been used for current-based fault diagnosis [17].

The drive current variation is affected not only by coil resistance but also by magnetic circuit states and equivalent inductance changes. Previous studies have shown that the inductance characteristics of electromagnetic actuators are sensitive to magnetic field conditions and motion positions [20]. Therefore, the equivalent flux-linkage variation is constructed according to the relationship among the driving voltage, resistance voltage drop, and current:

(20) $$\psi_{\mathrm{eq}}(t)=\int_{0}^{t}[U_{ref}-R_{\mathrm{ref}}i(\tau )]d\tau$$

where i represents the filtered drive current. $U_{ref}$ denotes the reference driving voltage. According to the measured results, the driving voltage remains approximately within 23.7–24 V during pulse excitation, and $U_{\mathrm{ref}}$ = 24 V is therefore adopted. $R_{\mathrm{ref}}$ represents the equivalent coil resistance under the normal state and is set to 5.3 Ω.

Based on the equivalent flux-linkage variation, the flux-linkage change at the end of stage I ($\Delta \psi_{eq}^{Ι}$) and the equivalent inductance variations in stages II ($L_{eq}^{\mathrm{II}}$) and III ($L_{eq}^{\mathrm{III}}$) are extracted to form three equivalent electromagnetic response features. Since a unified reference voltage and resistance are used, these features describe relative variations among different states rather than directly measured flux-linkage and inductance parameters.

Finally, the eight-dimensional stage macro features, three-dimensional stage dynamic features, and three-dimensional equivalent electromagnetic response features are combined to form the 14-dimensional feature vector:

(21) $$x={\left[\tau_{1},\tau_{2},k_{1},k_{2},k_{3},I_{1},I_{2},I_{3},a_{1},a_{2},a_{3},\Delta \psi_{eq}^{Ι},L_{eq}^{\mathrm{II}},L_{eq}^{\mathrm{III}}\right]}^{T}$$

### 4.3. GLR-CrossNet Classification Network

**Figure 17:**
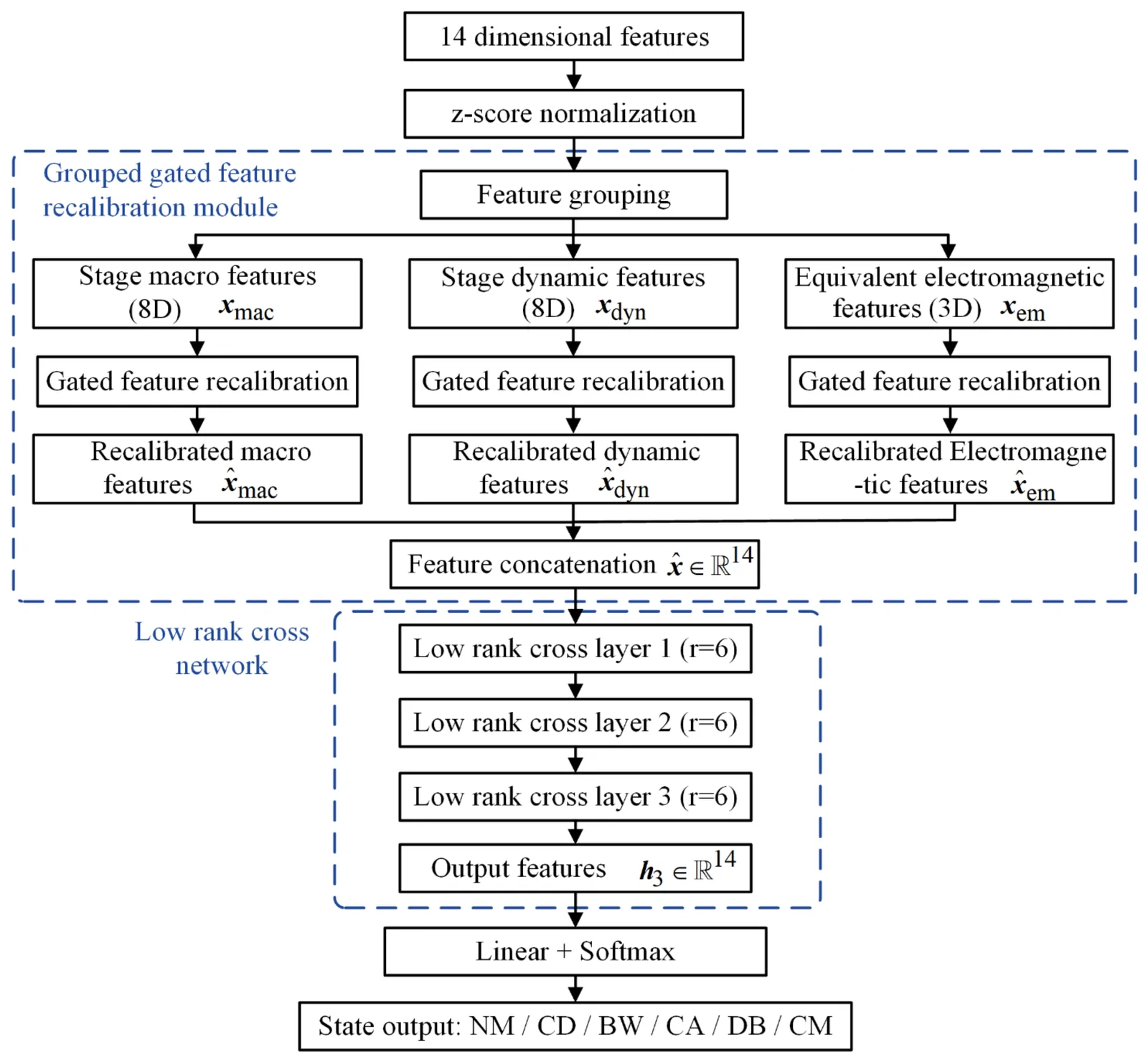
Overall architecture of GLR-CrossNet.

GLR-CrossNet consists of grouped gated feature recalibration and low-rank cross classification, with the overall architecture shown in Figure 17. The 14-dimensional input features are divided into three groups according to stage macro features, stage dynamic features, and equivalent electromagnetic response features, containing eight, three, and three dimensions, respectively. Each group is independently recalibrated by a gated unit. The processed features are then concatenated into a 14-dimensional vector and fed into the low-rank cross network for feature interaction modeling.

**Figure 18:**
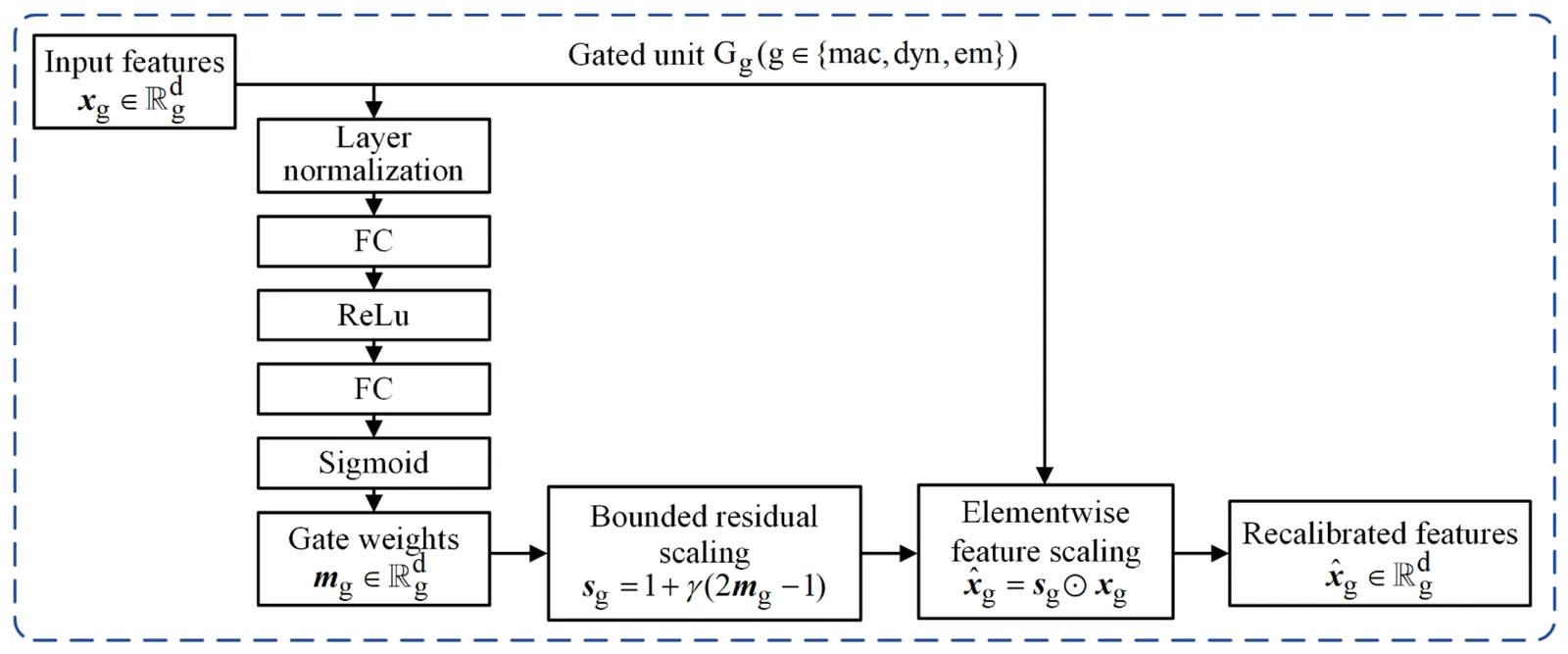
Grouped gated feature recalibration structure.

The grouped gated feature recalibration structure is shown in Figure 18. For the g-th input feature group $x_{g}$, the corresponding gate coefficients are obtained through layer normalization and feature mapping:

(22) $$m_{g}=\sigma [W_{g,2}\varphi (W_{g,1}\mathrm{LN}(x_{g})+b_{g,1})+b_{g,2}]$$

where LN(⋅) denotes layer normalization, φ(⋅) represents the ReLU activation function, and σ(⋅) is the Sigmoid function. The gated output is applied to the original group features after bounded scaling:

(23) $$s_{g}=1+\gamma (2m_{g}-1)$$

(24) $${\hat{x}}_{g}=s_{g}⊙x_{g}$$

where γ represents the scaling coefficient and ⊙ denotes element-wise multiplication. The three gated units adopt the same structure with independently learned parameters.

The recalibrated features are concatenated to obtain the fused vector $h_{0}$. The low-rank cross module adopts the matrix cross and low-rank parameterization strategy of Deep & Cross Network V2 (DCN V2) [34], where the cross matrix of the l-th layer is represented as the product of two low-dimensional matrices:

(25) $$W_{l}=U_{l}{V_{l}}^{T}$$

The corresponding cross layer update is expressed as:

(26) $$h_{l+1}=h_{l}+h_{0}⊙[U_{l}({V_{l}}^{T}h_{l})+b_{l}]$$

**Figure 19:**
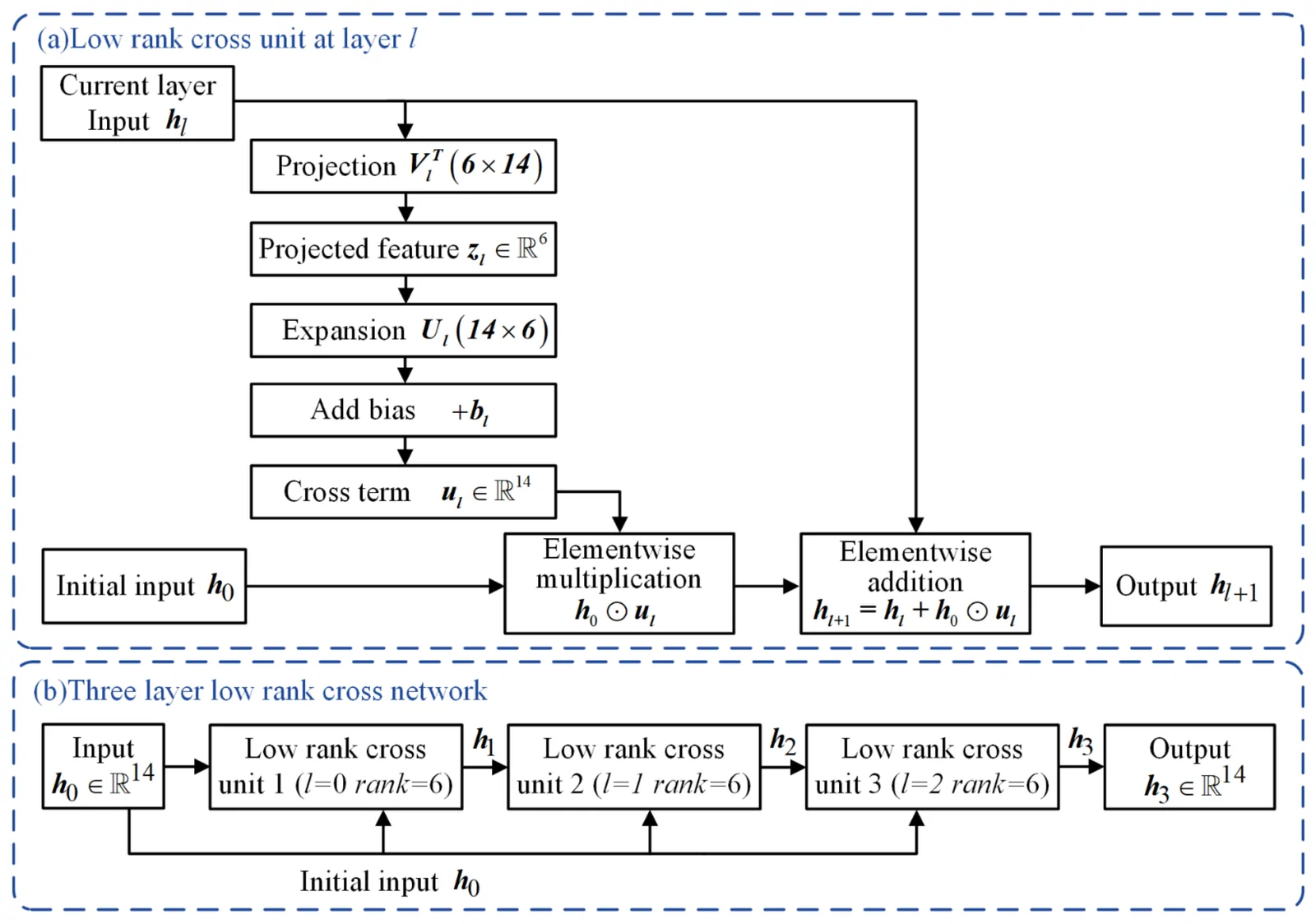
Three-layer low-rank cross network structure.

The model adopts a three-layer low-rank cross structure with the cross rank set to 6. The fused features are projected into a low-dimensional space and then remapped to the original feature dimension, while the initial input $h_{0}$ is retained in each cross layer for feature interaction. The overall structure is shown in Figure 19.

The output of the third layer $h_{3}$ is normalized and then input into a linear classifier. Softmax provides the prediction probabilities of the six states: NM, CD, BW, CA, DB, and CM. The main network configurations are summarized in Table 3.

### 4.4. Dataset Division and Model Training

The transient drive current dataset comprises 3600 valid actuation samples from 120 independent physical specimens across six states. For each state, 20 specimens were pre-pared, with 30 reversal responses collected from each specimen. The dataset composition for each partition is summarized in Table 4. To evaluate generalization across physical specimens, ten specimen-level partitions were generated using ten prespecified random seeds. In each partition, 12 specimens per state were randomly assigned to training, four to validation, and the remaining four to testing, corresponding to 2160, 720, and 720 current sequences, respectively. All sequences from the same specimen were kept in one subset, and all comparison models used the same partition within each run.

Before inputting the 14-dimensional feature vectors into the model, standard-score (z-score) normalization was performed:

(27) $$x_{q}^{*}=\frac{x_{q}-\mu_{q}}{\sigma_{q}}$$

where $\mu_{q}$ and $\sigma_{q}$ represent the mean and standard deviation of the q-th feature in the training set, respectively. The validation and test sets were transformed using the same statistical parameters.

GLR-CrossNet adopts cross-entropy as the classification loss and uses the Adam optimizer for parameter updating. The initial learning rate was set to $1\times 10^{-3}$, the mini-batch size was 64, and the maximum number of training epochs was 300. During training, the training samples were shuffled at each epoch, and a stepwise learning rate schedule was applied. The learning rate was reduced to 0.5 times its current value every 80 epochs, and L2 regularization was applied with a coefficient of $3\times 10^{-4}$. Validation loss was used for early stopping and model selection. Training was terminated when the validation loss did not improve for 40 consecutive epochs, and the model parameters with the lowest validation loss were retained.

To assess robustness to specimen-level partitioning, the model was trained using 10 random seeds, each corresponding to a newly generated specimen-level split, while all other training hyperparameters were kept identical. The mean and standard deviation of test Accuracy and Macro-F1 were calculated across runs. For the main model comparisons, 95% confidence intervals for performance differences were obtained from 2000 class-stratified paired bootstrap resamples at the physical-specimen level.

## 5. Fault Identification Results and Online Validation

### 5.1. Identification Results of Six States

**Figure 20:**
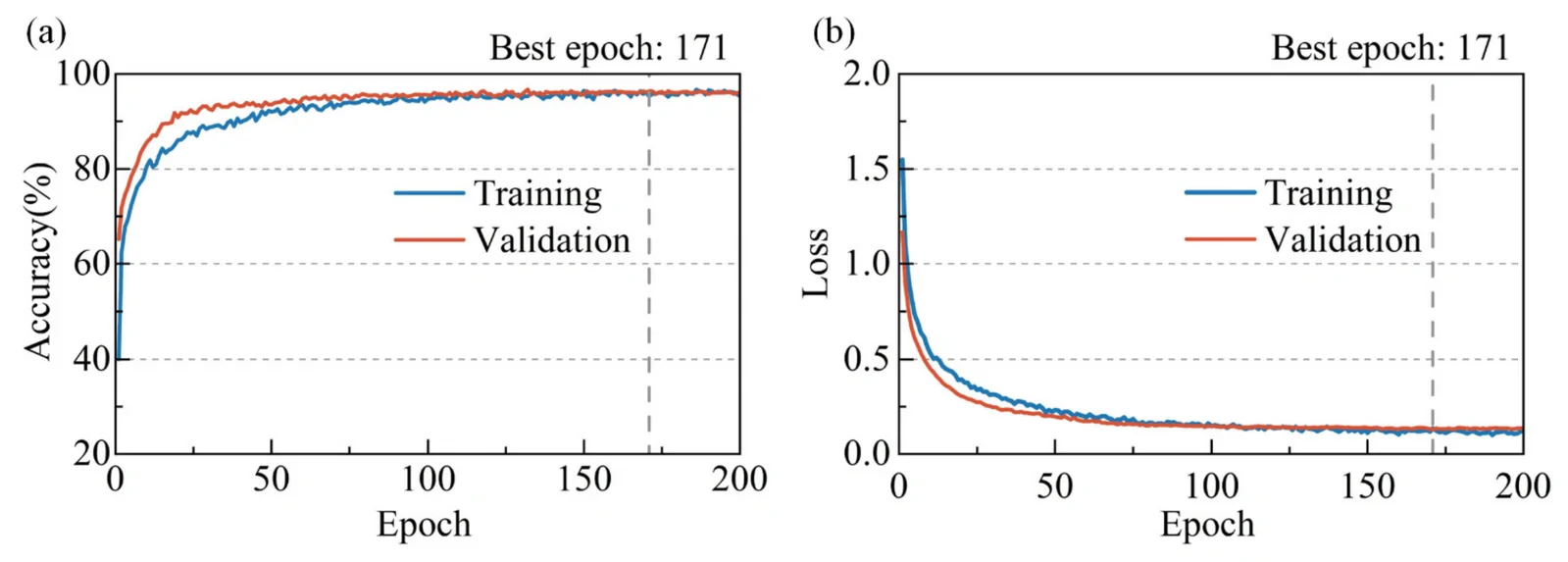
Training and validation curves of GLR-CrossNet: (**a**) Accuracy. (**b**) Loss.

The training and validation curves of GLR-CrossNet are shown in Figure 20. During the initial training stage, the loss decreases rapidly while the accuracy increases simultaneously. Both metrics gradually stabilize, and the dashed line marks the epoch with the lowest validation loss.

**Figure 21:**
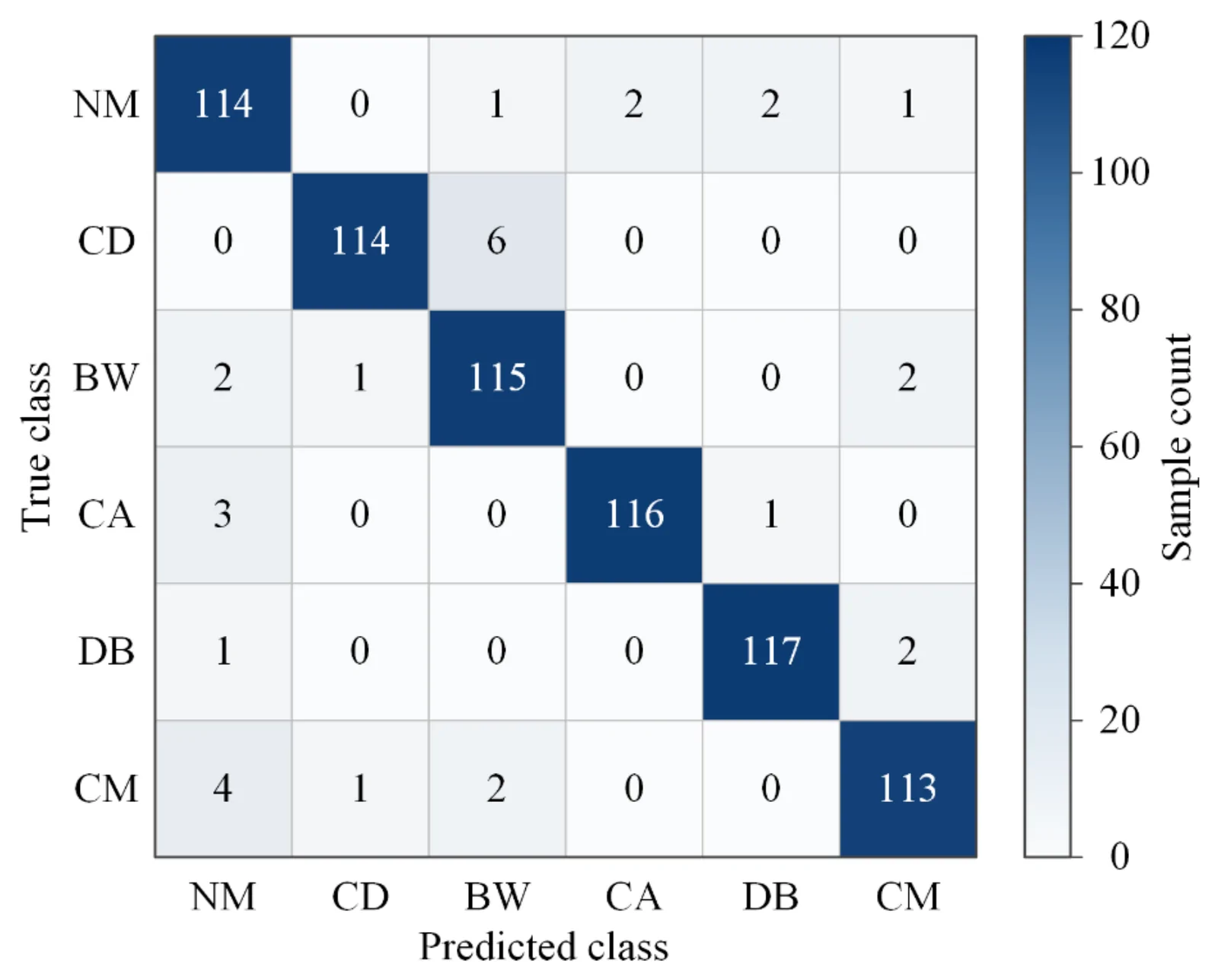
Confusion matrix of GLR-CrossNet on the independent test set.

The independent test set consists of four physical specimens from each of the six states that were not involved in training, containing a total of 720 drive current samples. For the single training run represented by the confusion matrix in Figure 21, GLR-CrossNet correctly identifies 689 samples, achieving an Accuracy of 95.69% and a Macro-F1 score of 95.71%.

Among the six states, DB achieves the highest Recall of 97.50%, whereas CM shows a relatively lower value of 94.17%. Six CD waveforms are misclassified as BW, accounting for the main classification errors in this state. The misclassification of CM is distributed among NM, BW, and CD. Cross recognition between DB and the other states is limited, and the main classification errors occur among several states with similar transient responses.

### 5.2. Comparison Experiments and Ablation Analysis

**Figure 22:**
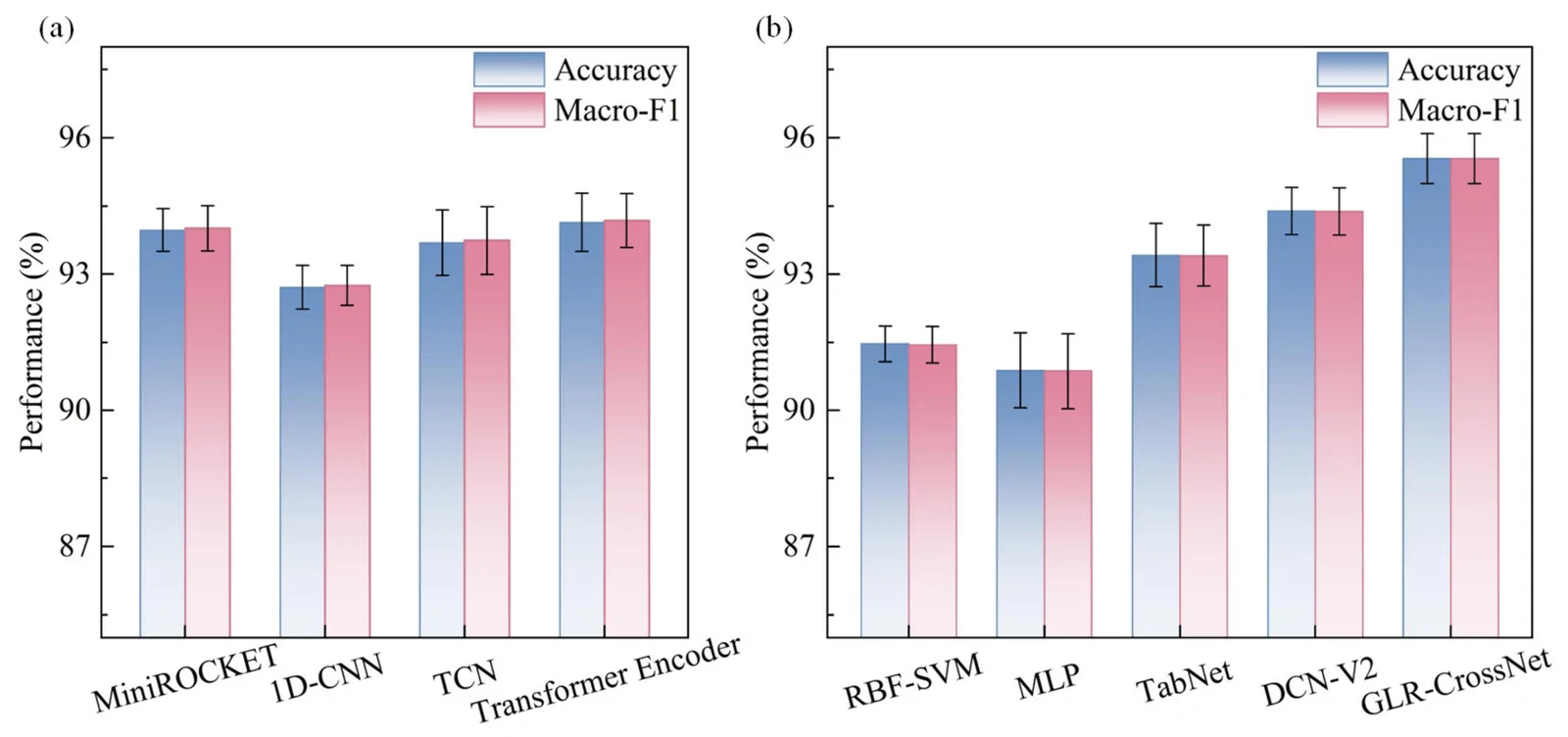
Performance comparison of different fault identification methods: (**a**) Raw current sequence input. (**b**) Fourteen-dimensional feature input. Bars represent the means over ten repeated runs, and error bars indicate ±1 sample standard deviation.

**Figure 23:**
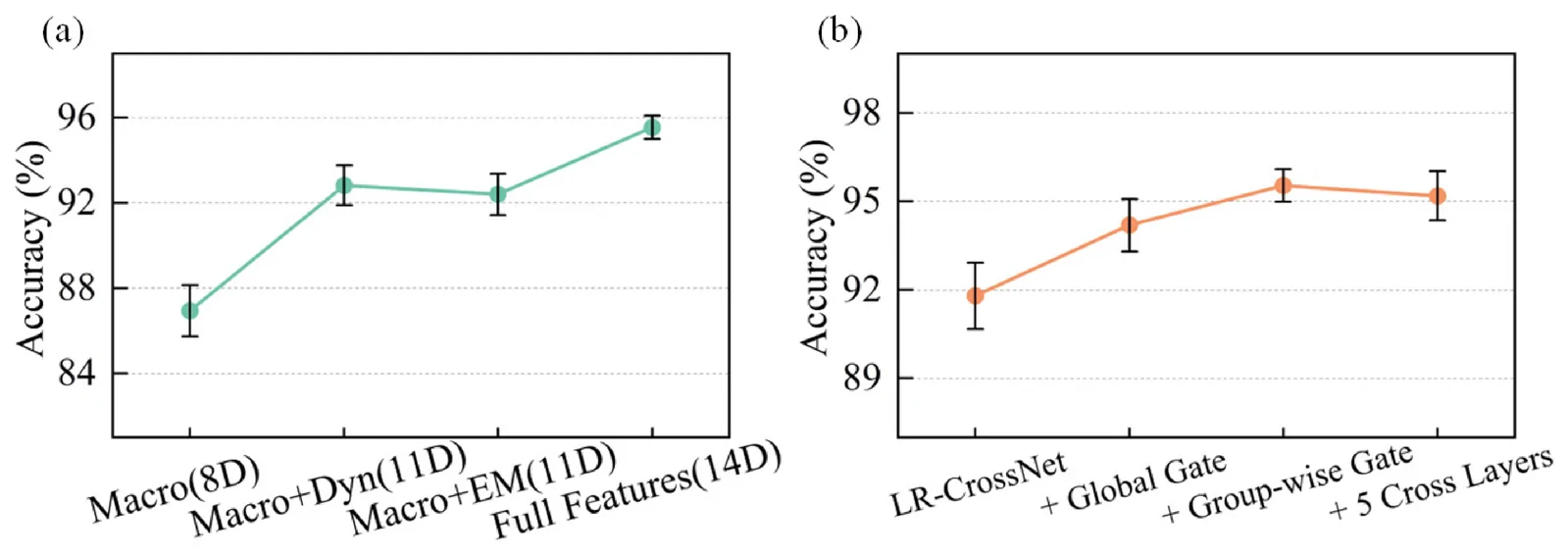
Ablation experiment results of GLR-CrossNet: (**a**) Feature combination ablation. (**b**) Model structure ablation.

To compare identification performance under different input forms and classification methods, MiniROCKET, 1D-CNN, TCN, Transformer Encoder, RBF-SVM, MLP, TabNet, and DCN-V2 were selected. Following the data partitioning protocol in Section 4.4, each model was trained under ten fixed random seeds, with each seed corresponding to one specimen-level data partition. Within each partition, all models used the same training, validation, and test specimens. The values plotted in Figure 22 and Figure 23 represent the means over ten runs, and the error bars indicate ±1 sample standard deviation. For the methods using the 161-point raw current sequence as input, all four models achieved relatively high identification performance, with Transformer Encoder performing best at an Accuracy of 94.14 ± 0.64%. With the 14-dimensional state features, GLR-CrossNet achieved the highest performance in this group, with an Accuracy and Macro-F1 of 95.54 ± 0.55% and 95.55 ± 0.56%, respectively, improving the Accuracy by 1.40 percentage points over Transformer Encoder. These results indicate that the 14-dimensional features substantially reduce the input dimension while retaining the main state information.

To analyze the contributions of the feature components and network structures, ablation experiments were conducted using the same ten experimental settings, as shown in Figure 23. Using only the eight-dimensional stage macro features resulted in an Accuracy of 86.94 ± 1.19%; adding the stage dynamic features increased it to 92.82 ± 0.94%, and the complete three-group feature set reached 95.54 ± 0.55%. This indicates that the stage dynamic and equivalent electromagnetic response features provide complementary state information beyond the macro features.

In the structural ablation experiment, the basic LR-CrossNet achieved an Accuracy of 91.78 ± 1.12%, which increased to 94.19 ± 0.89% and 95.54 ± 0.55% after adding global gating and grouped gating, respectively. This suggests that grouped recalibration helps preserve differences among the feature groups during fusion. Increasing the number of cross layers from three to five reduced the Accuracy to 95.19 ± 0.84%, indicating that three cross layers are sufficient for the current 14-dimensional input.

The sensitivity of the boundary localization to these parameters was further evaluated by varying each parameter separately, as summarized in Table 5. The baseline configuration used λ_1_ = 0.50, λ_2_ = 0.23, a 13-point third-order SG window, and a three-point median window, with one parameter varied at a time while the remaining settings were kept unchanged. Deviations of λ_1_ and λ_2_ from the baseline reduced Accuracy by up to 1.19 and 1.37 percentage points, respectively. An 11-point SG window reduced Accuracy by 2.41 percentage points, whereas a 15-point window caused a smaller decrease of 0.48 percentage points. The largest change occurred with a one-point median window, for which Accuracy decreased to 89.32 ± 2.48%. A one-point window effectively performs no median filtering and therefore cannot suppress isolated spikes, whereas an excessively large window may weaken short-duration variations near the stage boundaries. The 13-point SG and three-point median windows therefore provide a practical balance between noise suppression and preservation of stage transitions under the present conditions.

In addition, three, 12, and 47 boundary localization failures occurred at λ_1_ = 0.60, an 11-point SG window, and a one-point median window, respectively, based on all 3600 drive-current sequences per configuration. Localization failures in the test set were counted as identification failures.

To further assess the stability of the performance differences between the principal models, a paired bootstrap analysis was conducted with 2000 resamples, stratified by fault state, with individual selector specimens serving as the resampling units, as summarized in Table 6. The same resampled specimens were used to calculate the performance differences between GLR-CrossNet and each comparator model. The 95% confidence intervals of both Accuracy and Macro-F1 differences remained entirely above zero in both comparisons, indicating that the performance advantage of GLR-CrossNet remained stable under resampling at the individual-specimen level. The positive differences relative to DCN-V2 indicate better classification performance under the same 14-dimensional feature input, while the comparison with Transformer Encoder further shows that the proposed 14-dimensional state features combined with GLR-CrossNet achieve higher overall identification performance than classification using the raw current sequence.

### 5.3. Computational Complexity and Controller Deployment

Table 7 compares the computational complexity of the identification models. The 1D-CNN, TCN, and Transformer Encoder models, which take the 161-point raw current sequence as input, require approximately 1.34–2.67 million MACs per forward pass. In comparison, the classifiers using the 14-dimensional state features require only 752–12,608 MACs. Representing the raw current sequence with 14 features reduces the classifier input dimension by 91.3%, allowing classification without directly processing the full current sequence. However, the two groups also differ in network architecture, so their complexity differences reflect the combined effects of input representation and classifier design.

A comparison between GLR-CrossNet and DCN-V2, both using the same 14-dimensional input, further illustrates the effect of network design on computational complexity. GLR-CrossNet contains only 884 trainable parameters, requires 752 MACs per forward pass, and occupies approximately 3.45 KiB of FP32 weight storage. Compared with DCN-V2, its parameter count, MACs, and weight storage are reduced by 77.6%, 80.3%, and 77.7%, respectively. These reductions arise not only from low-rank factorization but also from the limited number of cross layers and the compact classification head. For each cross matrix, rank-6 factorization reduces the number of weights from 196 in a full 14 × 14 matrix to 2 × 14 × 6 = 168, a reduction of 14.3%. The network also uses three cross layers and a compact classification head. The structural ablation showed no further improvement in identification accuracy when the number of cross layers was increased to five, indicating that the additional depth did not yield a corresponding performance gain. Grouped gating, in contrast, recalibrates the three feature groups separately to improve feature fusion. Together, these design choices keep the model and its computational cost small.

GLR-CrossNet was further deployed on a GD32F303ZET6 controller operating at 120 MHz. As shown in Table 8, feature extraction took 2.76 ms, network inference took 0.18 ms, and the measured end-to-end identification latency was 3.11 ms, including approximately 160 μs for current acquisition and control. The final firmware occupied 15.31 KiB of Flash, with a peak RAM usage of 9.72 KiB. Feature extraction accounted for 93.9% of the combined feature extraction and inference time, indicating that front-end feature computation, rather than network inference, dominates the processing latency.

### 5.4. Continuous Operation Validation on Circular Knitting Machine

**Figure 24:**
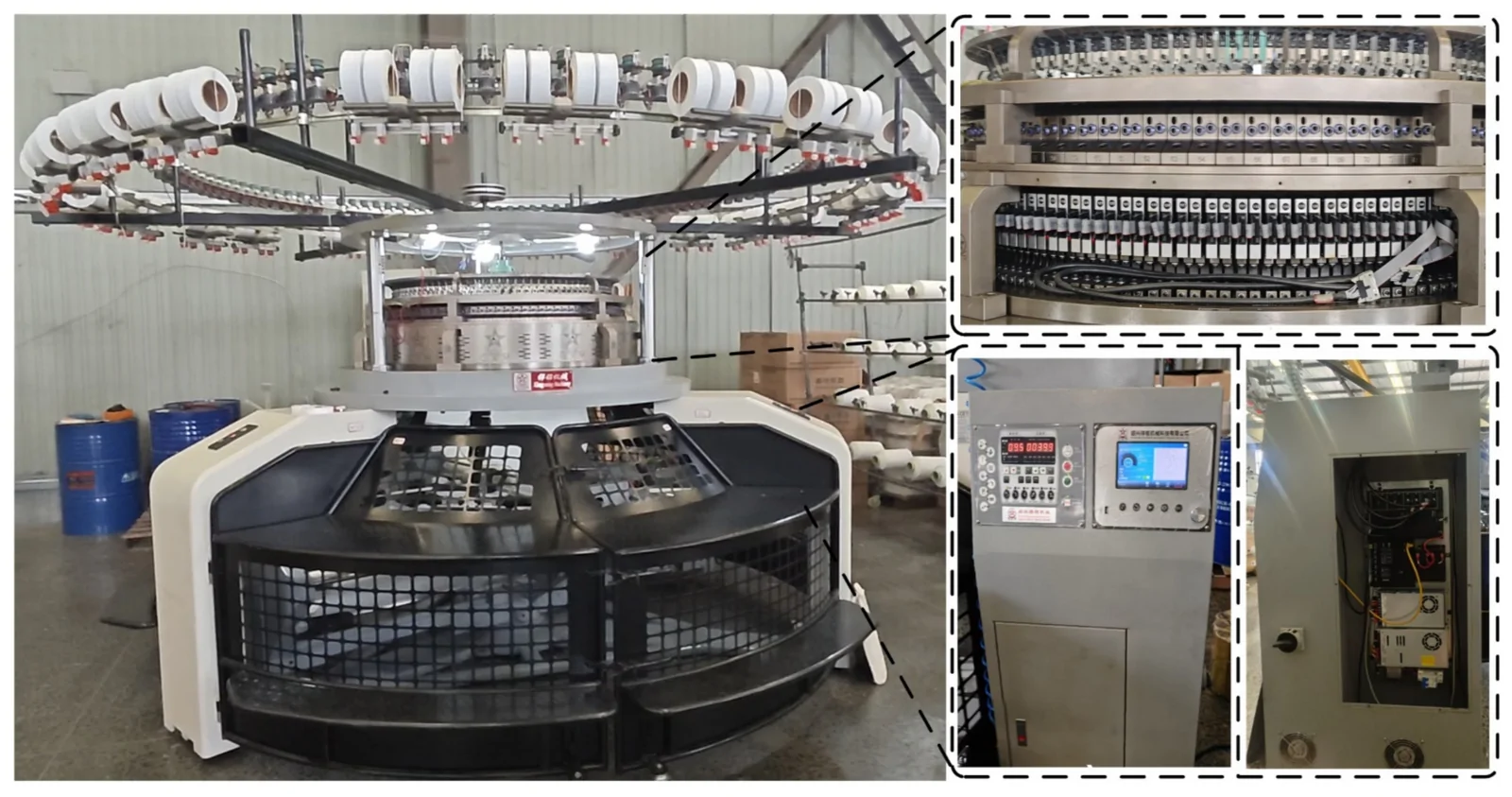
Online fault identification test platform on the circular knitting machine.

Continuous operation validation was conducted at room temperature on a circular knitting machine equipped with 144 magnetic latching needle selectors, as shown in Figure 24. Each selector contains 16 independent selector blade positions, giving a total of 2304 positions across the machine. The selector control terminal acquires the drive current, performs state identification, and uploads the confirmed state and blade position information to the main controller.

**Figure 25:**
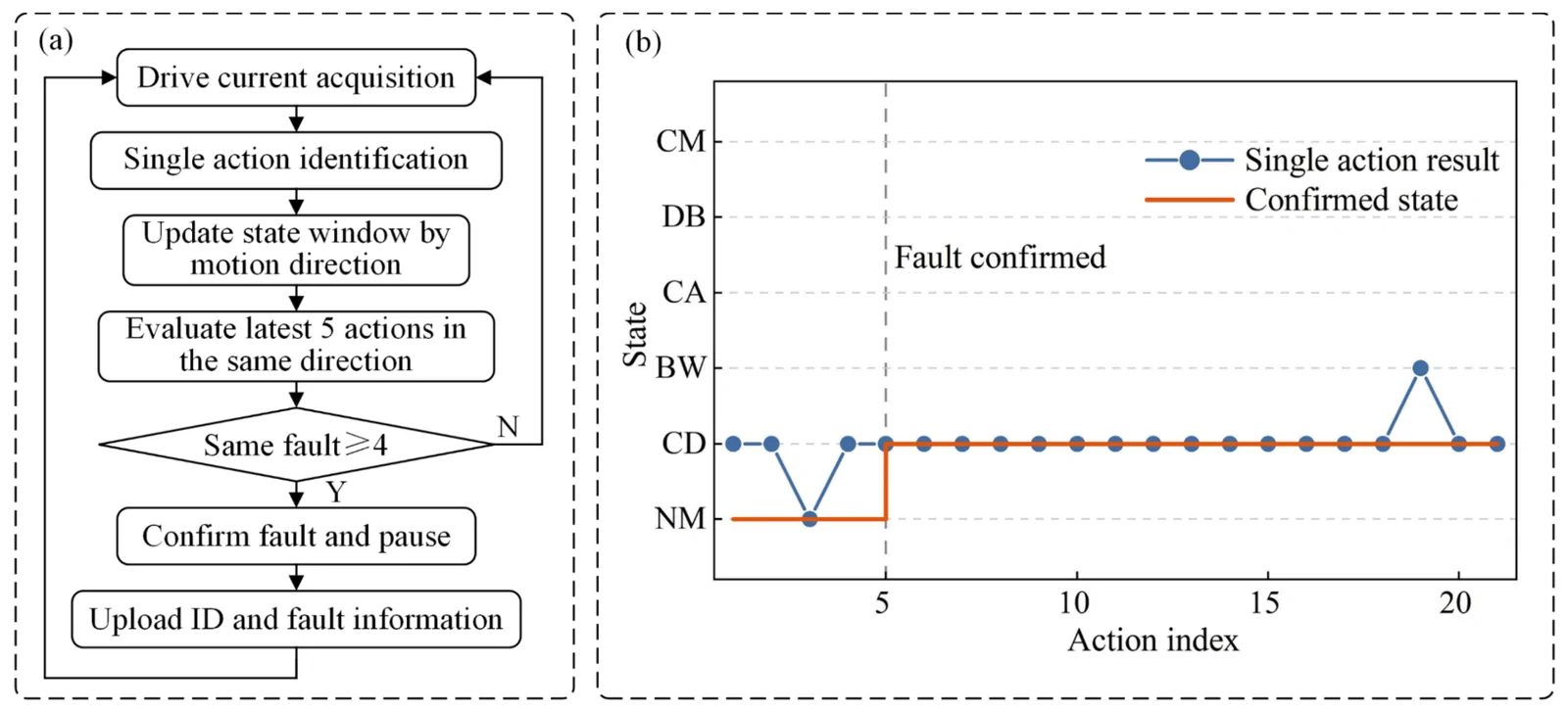
Continuous operation fault confirmation and identification results: (**a**) Fault confirmation process based on a sliding window. (**b**) Typical identification sequence during continuous operation.

Because some fault states exhibit different current responses depending on motion direction, separate state windows were maintained for each direction. A fault was confirmed when the same abnormal state was identified in at least four of the latest five actions in the same direction. Figure 25 illustrates the sliding-window confirmation procedure and a representative identification sequence during continuous operation.

The continuous operation results are summarized in Table 9. The NM state covered all 2304 blade positions, with 115,200 valid actuations recorded across the 144 selectors, corresponding to an average of 800 actuations per selector. The accuracy of a single identification was 94.80%. Although 5991 single identification errors occurred, no false alarms were recorded after window-based confirmation. For the five fault states, 100 blade positions were tested over 6000 valid actuations. The overall single identification accuracy was 94.20%, and fault confirmation required an average of 4.29 actions in the same direction, with no false alarms recorded.

The difference between single identification errors and final false alarms indicates that maintaining separate windows for each motion direction helped prevent occasional classification errors from affecting the final fault decision.

## 6. Conclusions

This study investigated the transient drive current responses of magnetic latching needle selectors under different fault states through electromechanical response analysis, specimen experiments, and continuous operation validation on a circular knitting machine. A multistage feature construction method and a lightweight fault identification model were developed and deployed on the control terminal. The main conclusions are as follows.

1. The transient drive current of the magnetic latching needle selector exhibits distinct three-stage response characteristics. Variations in the initial swing angle, magnetic circuit geometry, coil resistance, and core magnetic properties affect the stage boundaries, current growth trends, and amplitudes at key positions. The measured current waveforms of all six states retain the basic three-stage structure but differ in stage durations, local variations, and amplitude distributions, providing a basis for distinguishing different states from the transient drive current.
2. The combination of multistage state features and GLR-CrossNet enables lightweight identification of six states. The 14-dimensional features, comprising stage macro features, stage dynamic features, and equivalent electromagnetic response features, capture the main current differences among the states. Across ten runs with different specimen-level partitions, GLR-CrossNet achieved an Accuracy of 95.54 ± 0.55% and a Macro-F1 of 95.55 ± 0.56%, with only 884 trainable parameters. Comparison and ablation experiments showed that stage dynamic information, equivalent electromagnetic responses, and grouped gating all contributed to identification performance.
3. The proposed method enables millisecond-level state identification on the control terminal and continuous monitoring on the circular knitting machine. GLR-CrossNet requires only 752 MACs per forward pass, with an end-to-end identification latency of 3.11 ms. During continuous operation, five fault states were evaluated across 100 selector blade positions and 6000 valid actuations, achieving a single-action identification accuracy of 94.20%. With direction-specific state windows, faults were confirmed after an average of 4.29 actions in the same direction. No false alarms were recorded during 115,200 valid actuations under the NM state.

The experimental validation was mainly conducted at room temperature under the specified driving conditions. Temperature variations may affect coil resistance, driver response, and core magnetic properties, leading to changes in transient drive current features that may resemble those of certain fault states. The current method does not incorporate explicit temperature compensation, and its identification performance across different temperatures remains to be evaluated. Future work will examine performance under varying temperatures and operating conditions and investigate temperature compensation and training with multi-temperature data to improve adaptability under variable operating conditions.

## Figures and Tables

**Table 1:** Four parameters and perturbation settings.

Parameter	Normal State	Perturbation Level
Initial swing angle $\theta_{0}$	0°	−2°, −1°, +1°, +2°
Geometric offset Δx	0 mm	0.75, 1.50, 2.25, 3.00 mm
Equivalent coil resistance $R_{c}$	$R_{c0}$ = 5.30 Ω	5.57 Ω, 5.83 Ω, 6.10 Ω, 6.36 Ω
Saturation magnetization $M_{s}$	$M_{s0}$ = 1.90 × 10^6^ A/m	0.95 $M_{s0}$, 0.90 $M_{s0}$, 0.85 $M_{s0}$

**Table 2:** Six specimen states, actuation behaviors, and determination criteria.

State	Actuation Behavior	Determination Criterion
Normal state (NM)	Stable actuation	Intact structure, with coil resistance and core magnetic response within normal ranges
Coil degradation (CD)	Delayed actuation	Abnormally increased coil resistance
Baffle wear (BW)	Delayed actuation	Baffle wear and limit position offset, with an increased initial swing angle
Core aging (CA)	Delayed actuation	Reduced peak axial magnetic flux density at the core end under the same excitation
Debris blockage (DB)	Early actuation	Iron debris interferes with normal return, causing an offset of the initial blade position
Coil misalignment (CM)	Incomplete movement	Abnormal relative position of the coil, altering the magnetic circuit geometry

**Table 3:** Network configuration of GLR-CrossNet.

Component	Configuration
Input feature dimensions	14 (8/3/3)
Gate hidden dimensions	8/3/3
Gating coefficient	γ=0.35
Gating scale range	0.65–1.35
Low-rank cross network	3 layers, r = 6
Classification head	LayerNorm → FC (14→6) → Softmax
Output classes	6 (NM, CD, BW, CA, DB, CM)

**Table 4:** Composition of the six state specimens and datasets.

State	Specimens	Sequences per Specimen	Total Sequences	Training	Validation	Test
NM	20	30	600	12/360	4/120	4/120
CD	20	30	600	12/360	4/120	4/120
BW	20	30	600	12/360	4/120	4/120
CA	20	30	600	12/360	4/120	4/120
DB	20	30	600	12/360	4/120	4/120
CM	20	30	600	12/360	4/120	4/120
Total	120	30	3600	72/2160	24/720	24/720

**Table 5:** Sensitivity analysis of boundary localization parameters.

Parameter Setting	Accuracy (%)	Macro-F1 (%)	Boundary Failures (n)
Baseline	95.54 ± 0.55	95.55 ± 0.56	0
$\lambda_{1}$ = 0.40	94.40 ± 1.33	94.41 ± 1.33	0
$\lambda_{1}$ = 0.60	94.35 ± 1.09	94.39 ± 1.12	3
$\lambda_{2}$ = 0.18	94.64 ± 1.07	94.65 ± 1.06	0
$\lambda_{2}$ = 0.28	94.17 ± 1.33	94.17 ± 1.32	0
SG window = 11	93.13 ± 1.79	93.28 ± 1.78	12
SG window = 15	95.06 ± 0.76	95.06 ± 0.76	0
Median window = 1	89.32 ± 2.48	89.61 ± 2.48	47
Median window = 5	94.58 ± 1.11	94.59 ± 1.10	0

**Table 6:** Paired bootstrap confidence intervals for performance differences between GLR-CrossNet and comparison models.

Comparison	ΔAccuracy	95%CI	ΔMacro-F1	95%CI
GLR-CrossNet–DCN-V2	+1.153	[0.361,1.972]	+1.153	[0.379, 1.985]
GLR-CrossNet–Transformer Encoder	+1.403	[0.611,2.181]	+1.371	[0.583, 2.152]

**Table 7:** Classification performance and model complexity of different identification models.

Model	Input	Parameters	MACs	FP32 Weight Size (KiB)
1D-CNN	161 points	292,838	2,668,576	1143.90
TCN	161 points	73,478	2,552,992	287.02
Transformer Encoder	161 points	29,894	1,344,608	116.77
MLP	14 features	3366	3136	13.15
TabNet	14 features	13,890	12,608	54.26
DCN-V2	14 features	3952	3808	15.44
GLR-CrossNet	14 features	884	752	3.45

Note: FP32 weight size is calculated from the number of trainable parameters, assuming 4 bytes per parameter. It excludes firmware code and runtime buffers. MACs refers to the number of multiply–accumulate operations required for one forward pass of the classifier; feature extraction is excluded for models using the 14-dimensional features.

**Table 8:** Deployment performance of GLR-CrossNet on the control terminal.

Feature Extraction	Network Inference	End to end Latency	Flash	Peak RAM
2.76 ms	0.18 ms	3.11 ms	15.31 KiB	9.72 KiB

**Table 9:** Continuous operation test results on the circular knitting machine.

State	Tested Selector Blades	Valid Actuations	Single Action Accuracy (%)	Single Action Misclassifications	False Alarms	Average Confirmation Actions
NM	2304	115,200	94.80	5991	0	—
CD	20	1200	94.92	61	0	4.25
BW	20	1200	93.83	74	0	4.30
CA	20	1200	94.17	70	0	4.30
DB	20	1200	95.58	53	0	4.20
CM	20	1200	92.50	90	0	4.40
All faults	100	6000	94.20	348	0	4.29

## Data Availability

The data presented in this study are available on request from the corresponding author.
